# APOBEC3C Suppresses Prostate Cancer by Regulating Key Molecules Involved in Cellular Inflammation, Cell Cycle Arrest, and DNA Damage Response

**DOI:** 10.3390/cancers18010170

**Published:** 2026-01-03

**Authors:** Zhongqi Pang, Jianshe Wang, Yidan Xu, Bo Ji, Minghua Ren, Beichen Ding

**Affiliations:** 1 Department of Urology, First Affiliated Hospital of Harbin Medical University, Harbin 150001, China; 2021020849@hrbmu.edu.cn (Z.P.); wangjianshe0406@hrbmu.edu.cn (J.W.); 2022020903@hrbmu.edu.cn (B.J.); 2 Department of Pancreatic and Biliary Surgery, First Affiliated Hospital of Harbin Medical University, Harbin 150001, China; 3 Department of Urology, Yinfeng (Jinan) Hospital, Jinan 250014, China; 4 Department of Cardiology, First Affiliated Hospital of Harbin Medical University, Harbin 150001, China; 2022020675@hrbmu.edu.cn

**Keywords:** APOBEC3C, prostate cancer, prognosis, immune microenvironment, inflammation

## Abstract

**Background:** Prostate cancer (PCa) is a prevalent malignancy with a rising incidence. Advanced PCa, often resistant to therapy, remains a major clinical challenge, underscoring the need to identify novel molecular drivers. **Methods:** Utilizing transcriptomic data from the TCGA and GEO databases, we identified *APOBEC3C (A3C)* as a key candidate through WGCNA, differential expression analysis, and LASSO regression. Its clinical relevance was assessed via Kaplan–Meier survival analysis. Then, we validated *A3C* expression patterns using immunohistochemistry and Western blot in normal and malignant prostate cell lines. The functional effects of *A3C* on proliferation, migration, and invasion and mechanisms of such were evaluated through in vitro gain- and loss-of-function assays (CCK-8, Ki67 staining, wound healing, Transwell, Western blot, etc.). **Results:** *A3C* was significantly downregulated in PCa, and this low expression strongly correlated with adverse clinicopathological features, including advanced T stage, higher Gleason scores, and worse survival. Bioinformatically, high *A3C* expression was associated with an activated anti-tumor immune microenvironment, characterized by enhanced CD8+ T cell infiltration, reduced M2 macrophage abundance, and upregulation of the immune checkpoint *CD40*. In vitro, *A3C* overexpression effectively suppressed PCa cell proliferation, migration, and invasion, while its knockdown promoted these malignant phenotypes. Mechanistically, *A3C* enhances the expression of the *STING1* and its downstream related molecules *Caspase-1*, *IL-18*, and *IL-1β*; upregulates DNA damage-protective genes (*GSTP1* and *GPX3*); and enhances the expression of cell cycle regulator *GAS1*. **Conclusions:** This study establishes *A3C* as a suppressor in PCa, which impedes tumor progression by regulating key molecules involved in cellular inflammation, cell cycle arrest, and DNA damage response.

## Simple Summary

Given the clinical challenge of advanced, therapy-resistant prostate cancer (PCa), this study aimed to identify novel molecular drivers. Using transcriptomic data from the TCGA and GEO databases, combined with WGCNA, differential expression analysis, and LASSO regression, *APOBEC3C (A3C)* was identified as a key candidate, whose downregulation in PCa tumors correlated with advanced T stage, higher Gleason scores, and poor survival. Bioinformatic analysis linked high *A3C* expression to an anti-tumor immune microenvironment (e.g., increased CD8+ T cell infiltration and reduced M2 macrophages). In vitro assays confirmed that *A3C* overexpression suppressed PCa cell proliferation, migration, and invasion, while its knockdown promoted these malignant phenotypes. Mechanistically, *A3C* enhances the expression levels of *STING1* and its downstream molecules, including *Caspase1*, *IL-18*, and *IL-1β*, upregulating DNA damage protective genes (*GSTP1* and *GPX3*) and enhancing cell cycle regulator *GAS1* expression. Collectively, this study establishes *A3C* as a PCa suppressor that impedes tumor progression via multiple key pathways.

## 1. Introduction

Prostate cancer (PCa) is recognized as the most common malignant tumor among men in Western countries, with relatively high incidence and mortality rates [1]. Data from 2022 reveals that, although the number of new PCa cases in China is lower than that in Western countries like the United States, both the number of deaths and the incidence or mortality ratio are higher than those in the United States [2]. Surgery, endocrine therapy, radiotherapy, chemotherapy, immunotherapy, and combined therapy are the most commonly used treatment strategies for PCa and significantly improve the prognosis of patients with PCa [3]. However, it is noteworthy that, regardless of how remarkable the initial treatment efficacy is, almost all patients with progressive PCa will eventually experience treatment failure over time, especially those with castration-resistant PCa [4,5]. Therefore, investigating novel therapy and therapeutic biomarkers is essential for further improving the prognosis of PCa patients.

Genetic heterogeneity is one of the key factors driving the initiation, progression, drug resistance, and metastasis of PCa. The current understanding is that genetic variations among tumor cells endow the tumor with the ability to adapt to the microenvironment, evade therapy, and sustain proliferation. To explore the genomic variations in the development of PCa, bioinformatics analysis of gene expression levels and clinical traits in tumors can be used to facilitate the effective identification of key genes involved in tumorigenesis [6,7]. In this study, by applying integrated bioinformatic methods, *APOBEC3C (A3C)* was screened and identified as a potential prognostic gene in PCa.

*A3C* belongs to the *Apolipoprotein B mRNA-editing enzyme catalytic polypeptide-like 3* (*APOBEC3*, *A3s*) family located on human chromosome 22, which is a group of cytidine deaminases known for their antiviral activity [8]. In humans, the A3s family comprises seven members (*A3A*, *A3B*, *A3C*, *A3D*, *A3F*, *A3G*, and *A3H*) that function by deaminating cytosine to uracil on single-stranded DNA or RNA. Among the A3s family, *A3A* and *A3B* have been extensively studied in the context of cancer, largely due to their well-characterized roles in promoting tumor mutagenesis [9,10]. Emerging evidence supports the role of A3C in urological malignancies. Studies have indicated that the *A3C* gene is universally overexpressed in clear cell renal cell carcinoma (ccRCC) [11] and that it can activate the NF-κB signaling pathway to promote the progression of ccRCC [12]. In contrast, the role of *A3C* in PCa remains unclear. Some research has identified *A3C* as an mRNA diagnostic biomarker in the peripheral blood for PCa [13]. Other research teams have demonstrated that patients with higher prostate-specific antigen (PSA) levels exhibit lower *A3C* levels [14], as well as different *A3C* expression levels in different clinical pathogenic types [15]. Despite these compelling clinical associations, the precise functional role of *A3C* in PCa cell biology and the molecular mechanisms underlying its action remain virtually unexplored.

In this study, we identify *A3C* as a prognostic gene in PCa by utilizing integrated bioinformatic methods and explore the functions of *A3C* in PCa tumor cells via experimental validation.

## 2. Materials and Methods

### 2.1. Data Download

First, transcriptome data (553 sets in total, including 52 normal samples and 501 PCa patient samples) and clinical information for 500 PCa patients were retrieved and downloaded from The Cancer Genome Atlas Program (TCGA, National Cancer Institute, Bethesda, MD, USA) database. Second, raw data files from the GSE200879 dataset (137 transcriptome datasets in total, including 9 normal samples and 128 PCa patient samples) were accessed and downloaded from the Gene Expression Omnibus (GEO, National Center for Biotechnology Information, Bethesda, MD, USA) database. Subsequently, the “sva” package and “limma” package, batch correction, and data merging were run on the TCGA and annotated GSE200879 transcriptome data in R (software version 4.2.1). A transcriptome integrated dataset (hereinafter referred to as the integrated dataset) with a total sample size of 690 (including 61 normal samples and 629 PCa patient samples) was then obtained for subsequent use.

Furthermore, we obtained the transcriptome data of GSE69223, which includes the transcriptome profiles of 15 PCa tissues and their corresponding adjacent non-tumor tissues.

### 2.2. Screening and Identification of Prognosis-Related Genes

Weighted Gene Correlation Network Analysis (WGCNA) was conducted on the integrated dataset using the “WGCNA package” in R for gene cluster screening. The gene cluster demonstrating the most prominent expression in PCa was selected; subsequently, the most clinically relevant PCa-associated genes within this cluster were filtered by establishing thresholds where module membership exceeds 0.8 and gene significance surpasses 0.5. Next, the “limma” package was utilized to identify differentially expressed genes (DEGs) between PCa patients and normal individuals in the integrated dataset, employing criteria where the false discovery rate (FDR) is less than 0.05 and the absolute value of log_2_ fold change (log_2_FC) is at least 2. Subsequently, the “venn” package was used to obtain the intersection of the most significantly expressed PCa-related genes and the DEGs. These intersecting genes were subjected to further filtering via the Least Absolute Shrinkage and Selection Operator (LASSO) regression model, yielding a set of relatively optimal prostate PCa-associated genes. Then, Receiver Operating Characteristic (ROC) curves were plotted to evaluate the sensitivity and specificity of these PCa-related genes in predicting PCa, and validation was conducted in the GSE69223 dataset.

### 2.3. Gene Functional Analysis

Initially, patients from the TCGA dataset were stratified into high- and low-expression cohorts according to the median expression value of *A3C*. Differential expression analysis between these two cohorts was then performed using cutoff criteria of FDR < 0.05 and |log_2_FC| ≥ 1. Following the identification of DEGs, functional exploration of *A3C* and inference of potential molecular mechanisms were conducted through single-sample Gene Set Enrichment Analysis (ssGSEA), Kyoto Encyclopedia of Genes and Genomes (KEGG) pathway analysis, and Gene Ontology (GO) enrichment analysis. Furthermore, an *A3C* co-expression gene network was built with correlation coefficient > 0.6 and *p* < 0.001, while the STRING database was used to construct an A3C protein–protein interaction (PPI) network.

### 2.4. Analysis of Tumor Immune Microenvironment

The immune scores of samples were computed using the “estimate” package to preliminarily investigate associations between A3C expression levels and the tumor immune microenvironment. Subsequently, the “cibersort” package was used to investigate the effects of A3C on immune cell infiltration and the expression of immune checkpoints.

### 2.5. Cell Lines

The human normal prostate stromal immortalized cell line WPMY-1 was purchased from Procell Biotechnology Company (Wuhan, China). The human primary prostate cancer cell line 22RV-1, the brain metastatic cell line DU145, and the bone metastatic cell line PC-3 were obtained from the Cell Bank of the Chinese Academy of Sciences in Shanghai. WPMY-1 cells were cultured in DMEM; 22RV-1 and DU145 cells were cultured in RPMI-1640 medium; and PC-3 cells were cultured in F12K medium. All basal culture media were supplemented with 10% fetal bovine serum and 1% penicillin–streptomycin. All the cell lines used in this study were authenticated by cell source institutions and were confirmed to be free from mycoplasma.

### 2.6. Antibodies

All antibodies were used in this study by following the manufacturer’s instructions: Anti-b-Tubulin (AC008, Abclonal, Wuhan, China), anti-APOBEC3C/PBI (ab181356, Abcam, Shanghai, China), anti-Caspase-1 (ab207802, Abcam, Shanghai, China), anti-IL1b (ab216995, Abcam, Shanghai, China), anti-IL18 (ab243091, Abcam, Shanghai, China), anti-STING1 (ab181125, Abcam, Shanghai, China), anti-CD40 (84406-1-RR, Proteintech, Wuhan, China), anti-GAS1(67181-1-Ig, Proteintech, Wuhan, China), anti-GPX3 (13947-1-AP, Proteintech, Wuhan, China), anti-GSTP1 (86708-1-RR, Proteintech, Wuhan, China), anti-Ki67 (ab92742, Abcam, Shanghai, China), rabbit anti-Goat secondary antibody (ZB-2301, ZSGB-bio, Beijing, China), and fluorescent secondary Cy3 rabbit anti-Goat IgG (H + L) antibody (K1215, ApexBio, Shanghai, China).

### 2.7. Plasmids and siRNA

The A3C overexpression plasmid pEX-Z7698-M98 and its corresponding empty vector were both purchased from GeneCopoeia (Guangzhou, China). The A3C gene knockdown small interfering RNA (siRNA) was purchased from Shanghai Hanbio (Shanghai, China). hs-APOBEC3C-si1, F: GCUCAGUUGUCUCCUGGAATT, R: UUCCAGGAGACAACUGAGCTT. hs-APOBEC3C-si2, F: GACCCAUUGUCAUGCAGAATT, R: UUCUGCAUGACAAUGGGUCTT. hs-APOBEC3C-si3, F: GGAGAUCAUGGACUAUGAATT, R: UUCAUAGUCCAUGAUCUCCTT. NC, F: UUCUCCGAACGUGUCACGUTT, R: ACGUGACACGUUCGGAGAATT.

### 2.8. Cell Transfection

The protocol for overexpression plasmids was consistent with that for siRNA, differing only in grouping. Briefly, PCa cell lines were seeded into 6-well plates and cultured in complete medium until cell confluence reached 80–90% (for transfection preparation); two hours before transfection, the complete medium in each well was replaced with 1.5 mL serum-free Opti-MEM, and the plates were placed in a 37 °C incubator. For grouping, plasmid-transfected cells were divided into a control group (negative control, NC) and an overexpression (OE) group, while siRNA-transfected cells were first divided into blank, NC, Lipofectamine 2000 (Lipo2000, Thermo Scientific, Shanghai, China), siRNA1, siRNA2, and siRNA3 groups to verify transfection efficiency, with subsequent functional studies using the NC group and the selected effective siRNA group. For transfection operation, 10 μL of plasmid/siRNA was mixed with 240 μL of Opti-MEM and incubated for 5 min, and separately, 5 μL of Lipo2000 was mixed with 245 μL of Opti-MEM at RT and incubated for 5 min. The two solutions were then combined to form a 500 μL transfection mixture, incubated at RT for 10 min, and added to each well (total volume per well: 2 mL) at a temperature of 37 °C for 8 h. Then, the mixture was removed, 2 mL of antibiotic-free complete medium was added to each well for overnight culture, and transfection efficiency was initially determined based on its fluorescence ratio under a fluorescence microscope (for plasmids) or directly evaluated based on the target protein level (for siRNA), with a fluorescence ratio > 80% considered sufficient for subsequent experiments.

### 2.9. Western Blot

SDS-PAGE gels were prepared according to the kit instructions based on the target protein molecular weight. Samples were taken from −20 °C storage, heated at 95 °C for 5 min, and vortexed. Protein concentration was determined through BCA assay, with 20 μg of protein loaded per well; a 2 μL protein marker was added to lanes flanking the samples. Electrophoresis buffer was used for electrophoresis at 80 V constant voltage for 30 min and then 120 V for approximately 1 h. Transfer buffer was chilled on ice. After electrophoresis, the resolving gel was transferred to a transfer sandwich without drying, ensuring no bubbles between the gel and membrane (PVDF membrane, activated with methanol prior to use). The sandwich was placed in a transfer tank with sufficient chilled buffer, and proteins were transferred at 200 mA constant current for 2 h in an ice-water bath. TBST buffer and skim milk blocking solution were used post-transfer: membranes were washed twice with TBST (5 min each), blocked in blocking solution on a shaker for 2 h, and then washed three times with TBST (5 min each). Next, the membranes were incubated in primary antibodies overnight at 4 °C. After washing the membranes with TBST (5 min each, three times), the membranes were incubated in secondary antibodies on a shaker for 1 h at room temperature, and the protein bands were visualized through chemiluminescence (ECL), with exposure duration varying depending on the expression level. The grayscale values of target protein bands were normalized against the corresponding internal reference proteins. Subsequent statistical analyses were performed on the normalized data rather than the absolute grayscale values. Unprocessed blots can be found in the supplementary document (Supplementary Figure S6).

### 2.10. Cell Proliferation Assay

PCa cells 24 h post-transfection were trypsinized with 0.25% trypsin, collected into 15 mL centrifuge tubes, centrifuged at 1000 rpm for 3 min, washed twice with PBS, and resuspended in complete medium. The cell concentration was adjusted to 25,000 cells/mL using a hemocytometer. Then, 200 μL of cell suspension was seeded into each well of a 96-well plate with 3 replicate wells per group, and 4 plates were prepared to ensure consistent cell numbers. At 0, 24, 48, and 72 h after seeding, 10 μL of Cell Counting Kit-8 (CCK8, Beyotime, Shanghai, China) solution was added to each well, and absorbance at 450 nm was measured to analyze cell proliferation. Two-way ANOVA followed by Šídák multiple comparison test was used to analyze the results.

### 2.11. Cell Ki67 Staining Assay

PCa cells were collected into 15 mL centrifuge tubes, centrifuged at 1000 rpm for 3 min, washed twice with PBS, and fixed with 4% paraformaldehyde at room temperature for 10 min. After fixation, the cells were washed 3 times with PBS and then incubated in blocking–permeabilizing solution for 30 min at room temperature. Ki67 antibody was diluted according to its instructions. After permeabilization, the cells were transferred to 1.5 mL centrifuge tubes, centrifuged at 1000× *g* to remove the permeabilizing solution, washed 3 times with PBS, and then incubated with diluted Ki67 antibody solution with thorough mixing on a shaker at 4 °C overnight. The next day, the antibody solution was removed; subsequently, the cells were washed and then incubated with diluted fluorescent secondary antibody (protected from light) in a dark box for 1 h at room temperature. After incubation, the cells were centrifuged, the antibody solution was removed in the dark, the cells were washed three times, and then they were mixed thoroughly with 100 μL of PBS and 20 μL of DAPI solution. After 10 min, 10 μL of cell suspension was dropped onto a slide, covered with a coverslip, and imaged under a fluorescence imaging system at 10 × 20 magnification in a darkroom. We used ImageJ software (National Institutes of Health, USA, 1.53q) to perform quantitative analysis on the merged fluorescence images. The average expression level of Ki67 (Mean) in PCa cells was assessed by measuring the fluorescence intensity of Ki67 (red signal) within cells (Area), with relative fluorescence intensity calculated based on the mean value from the NC group (fold of NC).

### 2.12. Wound Healing Assay

At 24 h post-transfection, PCa cells were seeded into 6-well plates, and the wound healing assay was initiated when cell confluence reached over 90%. Three straight lines with as uniform width as possible were vertically scratched in each well using a 200 μL pipette tip along a sterile ruler to create cell-free wounds. The cells were washed twice with PBS and then cultured in serum- and antibiotic-free basal medium (medium containing 4% serum was used for siRNA-transfected cells). Images were captured at 0, 24, and 48 h after wounding under 10 × 4 magnification. The same imaging positions were maintained across different time points using external markers.

### 2.13. Cell Migration and Invasion Assays

For the migration assay, at 24 h post-transfection, PCa cells were first cultured in serum-free medium for 12 h, then trypsinized with 0.25% trypsin, washed twice with PBS, and resuspended in serum-free medium to a density of 2.5 × 10^5^ cells/mL. A 200 μL aliquot of the serum-free cell suspension was thoroughly mixed and added to the upper chamber of a Transwell insert; 500 μL of complete medium was added to the corresponding well of the 24-well plate. The insert was carefully placed into the well and incubated in a 37 °C cell culture incubator for 24 h. After 24 h, the cells on the serum-free side of the membrane were gently wiped off with a moist cotton swab. The insert was then fixed by submerging the serum-exposed side in 4% paraformaldehyde for 10 min, air-dried, and stained with crystal violet solution for 10 min, followed by gentle rinsing with water. After air-drying, images were captured under an inverted microscope at 10 × 20 magnification.

The invasion assay followed the same protocol as the migration assay, except that the Transwell insert was pre-treated with Matrigel before cell seeding.

### 2.14. Statistical Analysis

All the experiments were repeated at least three times at the biological level. Bioinformatics analyses were performed using R and Perl software (version 5.30.0.1-64 bit). Data from the Western blot and wound healing assays were quantified using ImageJ software (National Institutes of Health, Bethesda, MD, USA, version 1.53 q), followed by statistical analysis and visualization in GraphPad Prism 10. In particular, one-way analysis of variance (one-way ANOVA) was employed for comparisons across three groups. Two-way ANOVA was used to analyze the results including the temporal factor, while *t*-tests were utilized for between-group comparisons involving two groups. A *p*-value < 0.05 was considered statistically significant, denoted as follows: ns, not significant, * *p* < 0.05; ** *p* < 0.01; *** *p* < 0.001; **** *p* < 0.0001.

## 3. Results

### 3.1. Screening and Identification of A3C as a PCa-Related Gene

**Figure 1:**
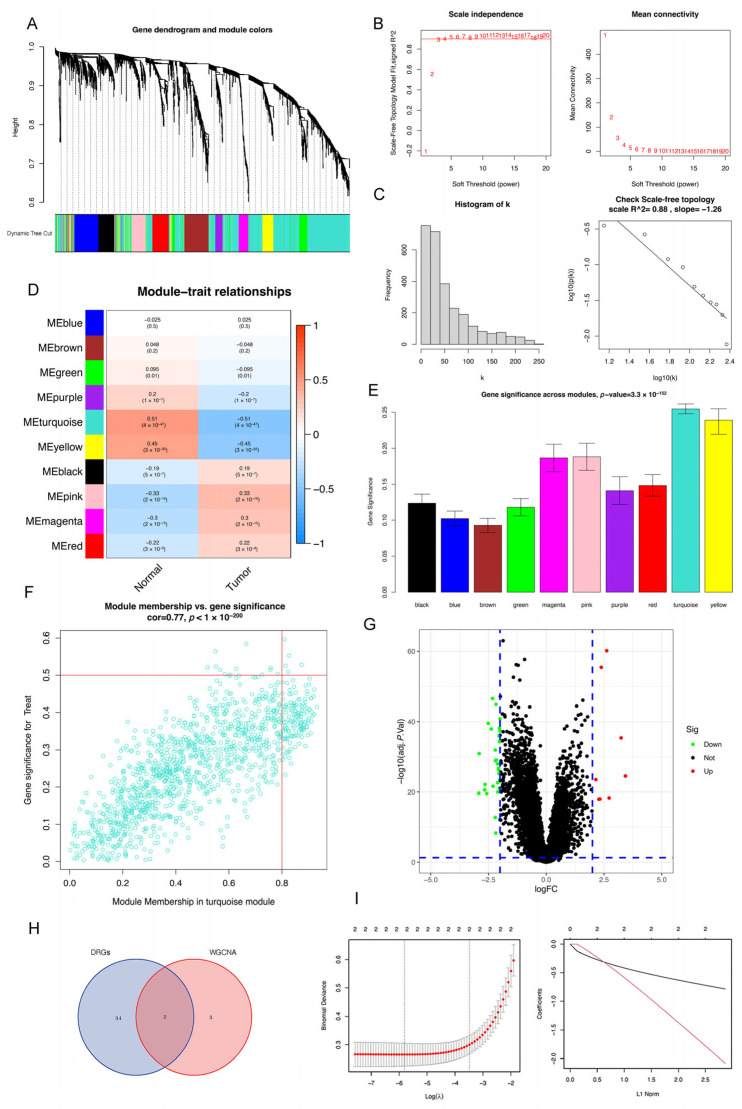
Gene screen and identification in PCa via bioinformatics analysis. (**A**) Dynamic trees of WGCNA. (**B**) Scale independence, numbers refer to values of soft-thresholding power. (**C**) Soft connectivity. (**D**) Relationships of gene modules between normal samples and tumor samples. (**E**) Gene significance across gene modules. (**F**) Module membership and gene significance in turquoise module, turquoise dots refer to genes and red lines refer to thresholds. (**G**) Volcano map of differentially expressed genes. (**H**) Venn map, blue circle refers to DRGs and red circle refers to WGCNA genes. (**I**) Results of 2 PCa-related genes in LASSO analysis.

To systematically identify key drivers of PCa pathogenesis, we employed a multi-step bioinformatic approach on an integrated transcriptome dataset (690 samples: 61 normal controls, 629 PCa patients). First, we performed WGCNA to uncover groups of functionally related genes. This constructed a scale-free co-expression network, which yielded 10 distinct modules (Figure 1A). Further analysis determined that a soft-thresholding power of β = 3 was optimal for balancing scale-free topology and mean connectivity (Figure 1B,C). Among these, the “Turquoise” module exhibited the strongest significant correlation with PCa status and the highest gene significance (Figure 1D,E). With the thresholds set at greater than 0.8 for module membership and above 0.5 for gene significance, this module yielded five hub genes (*ANGPT1*, *AOX1*, *A3C*, *ASPA*, and *C2orf88*) that are critically associated with PCa (Figure 1F).

Subsequently, we identified DEGs between normal and PCa samples, applying thresholds of FDR < 0.05 and |log_2_FC| ≥ 2. This analysis yielded 36 DEGs (8 upregulated, 28 downregulated), which were visualized in a volcano plot (Figure 1G). Intersection of these 36 DEGs with the 5 hub genes from the WGCNA pinpointed two overlapping genes, *AOX1* and *A3C*, as high-confidence candidates (Figure 1H).

Finally, we utilized the integrated dataset to train a LASSO regression model and the two previously identified differentially expressed genes, *AOX1* and *A3C*, associated with PCa were incorporated into this model. The results show that both genes conformed to the LASSO regression criteria and demonstrated a negative correlation with PCa development, suggesting their potential tumor-suppressive roles (Figure 1I).

### 3.2. A3C Exhibits Robust Diagnostic Efficacy for PCa

We next evaluated the diagnostic potential of the two candidate genes, *AOX1* and *A3C*. In the integrated cohort, *A3C* demonstrated exceptional diagnostic power, with an area under the curve (AUC) of 0.956 (95% CI: 0.925–0.980), and *AOX1* also showed high efficacy (AUC = 0.938, 95% CI: 0.904–0.966) (Supplementary Figure S1A,B). This robust performance was validated in an independent external dataset (GSE69223), where A3C again achieved a higher AUC of 0.858 (95% CI: 0.693–0.982) compared with 0.769 for *AOX1* (95% CI: 0.564–0.920) (Supplementary Figure S1C,D). These results confirm that both genes are strong diagnostic biomarkers for PCa.

Despite the strong diagnostic performance of both genes, we focused subsequent mechanistic studies on *A3C*. While the role of *AOX1* in PCa has been previously established, the specific function and mechanism of *A3C* in this malignancy remain largely unexplored. Therefore, we selected *A3C* as the primary target for further functional investigation.

### 3.3. Low Expression Level of A3C Correlates with Poor Prognosis in PCa

**Figure 2:**
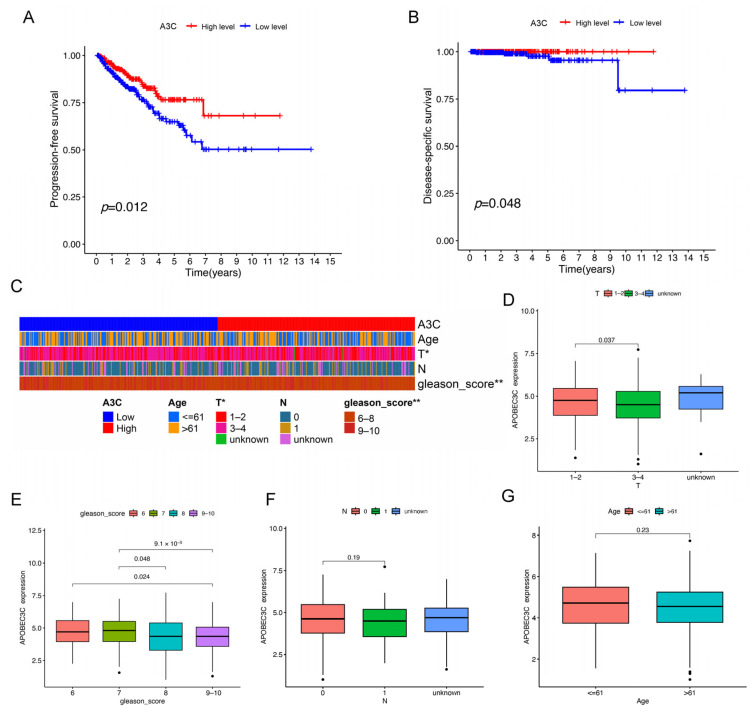
Low expression of A3C is correlated to poor prognosis of PCa. (**A**,**B**) Kaplan–Meier analysis for PFS and DSS of *A3C* in PCa. (**C**) Heatmap of correlation between *A3C* and clinical parameters. (**D**–**G**) The relationships between *A3C* expression and T stage, Gleason score, N stage, and age, dots refer to outlier samples. * *p* < 0.05, ** *p* < 0.01.

We subsequently assessed the clinical relevance of *A3C* expression using data from the TCGA database. A Kaplan–Meier analysis revealed that patients with low *A3C* expression (below the median) had significantly worse progression-free survival (PFS, *p* = 0.012) and disease-specific survival (DSS, *p* = 0.048) compared with those with high *A3C* expression (Figure 2A,B). Furthermore, low *A3C* expression was significantly associated with aggressive disease features, including higher Gleason scores and advanced T stage (Figure 2C–G). Collectively, these data unequivocally demonstrate that low *A3C* expression is a robust biomarker associated with advanced disease stage and poorer prognosis in PCa patients.

**Figure 3:**
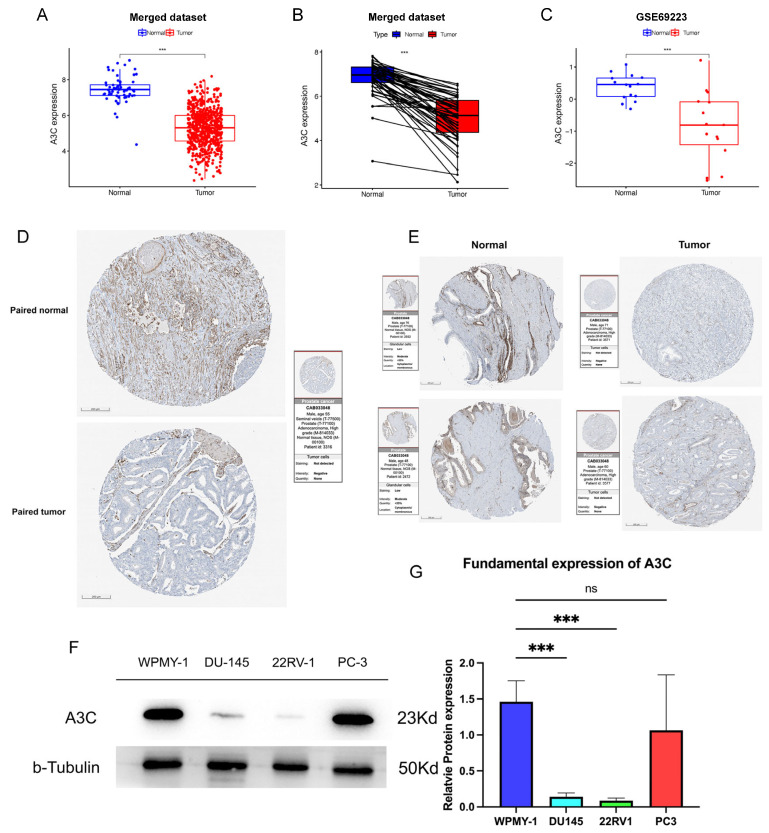
The expression of *A3C* is downregulated in prostate cancer (PCa). (**A**) Expression of *A3C* in merged dataset, unpaired *t*-test, two-tailed. (**B**) Expression of *A3C* in paired samples in merged dataset, paired *t*-test, two-tailed. (**C**) Expression of *A3C* in GSE69223, unpaired *t*-test, two-tailed. (**D**) Paired samples of immunohistochemistry. (**E**) Unpaired samples of immunohistochemistry. (**F**) Western blot analysis of basal A3C expression in PCa cells. (**G**) Grayscale analysis for plots in (**F**); data is presented as mean ± SD (*n* = 5 independent experiments). One-way ANOVA followed by Dunnett’s multiple comparison test. ns, not significant, *** *p* < 0.001.

Besides, an analysis of the integrated dataset revealed a significant reduction in *A3C* expression in PCa tissues compared with normal controls (Figure 3A). This downregulation was consistently observed in paired samples from the same dataset (Figure 3B) and was robustly validated in the independent GSE69223 cohort (Figure 3C).

We next sought to validate these findings at the protein level using immunohistochemical (IHC) data from the Human Protein Atlas (HPA). Consistent with our transcriptomic data, A3C protein was weakly expressed in adjacent non-tumor tissues but was virtually undetectable in matched PCa tissues (Figure 3D). It was further confirmed in two additional sets of unpaired IHC samples (Figure 3E), strongly supporting the downregulation of A3C at the protein level in PCa. To extend our investigation to in vitro models, we examined A3C expression in a panel of prostate cell lines. Western blot analysis confirmed significantly lower A3C expression in the orthotopic PCa cell line 22RV-1 and the brain metastasis-derived line DU145 compared to the normal prostate stromal cell line WPMY-1. The expression of A3C from the bone metastasis-derived PC-3 cell line showed comparable results to those of WPMY-1. These findings, consistent across three independent replicates (Figure 3F,G), indicate that A3C expression is frequently reduced in PCa cell lines.

### 3.4. Overexpression of A3C Inhibits Proliferation, Migration, and Invasion of PCa Cells

**Figure 4:**
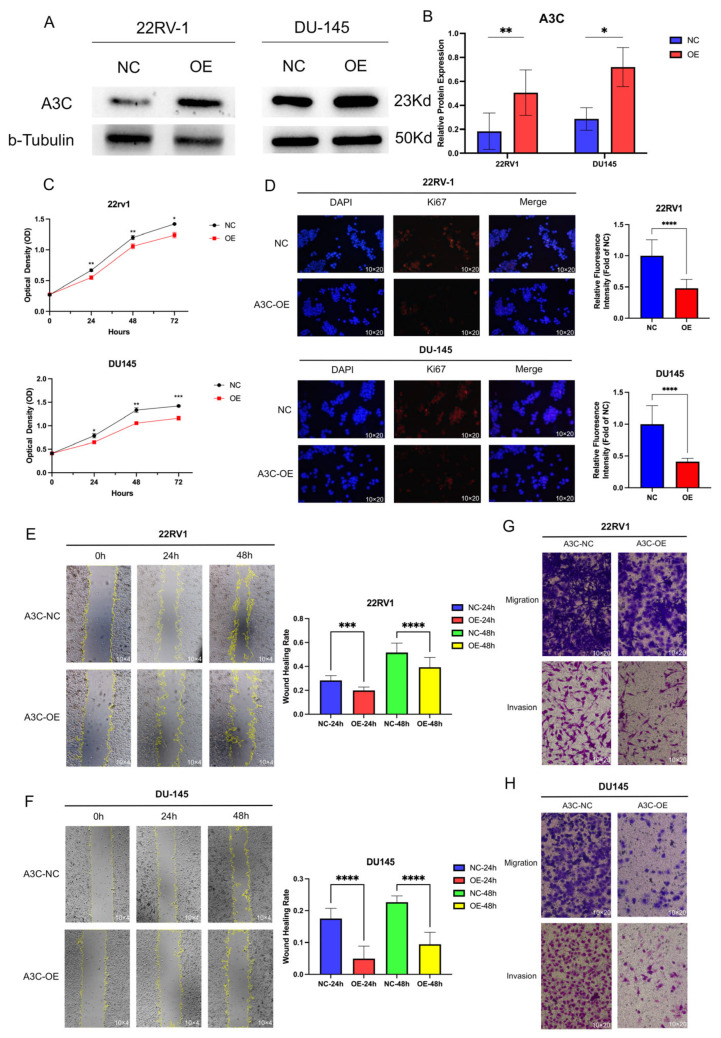
Overexpression of A3C inhibited the cell proliferation, migration, and invasion abilities of PCa cells. (**A**) Detection of A3C overexpression by Western blot. (**B**) Grayscale analysis for plots in (**A**). Data are presented as mean ± SD (*n* = 5 for 22RV1 and *n* = 4 for DU145, independent experiments), two-tailed unpaired *t*-test. (**C**) Results of CCK8. Data are presented as mean ± SD (*n* = 4 independent experiments), two-way ANOVA followed by Šídák multiple comparison test. (**D**) Immunofluorescent pictures of Ki67 and relative fluorescence intensity (fold of NC) in 22RV1 and DU145 cells, data are presented as mean ± SD (*n* = 3 independent experiments for each cell), two-tailed unpaired *t*-test. (**E**,**F**) Wound healing assay in 22RV1 and DU145, respectively. Data are presented as mean ± SD (*n* = 3 independent experiments), two-way ANOVA followed by Šídák multiple comparison test. (**G**) Transwell assay in 22RV1. (**H**) Transwell assay in DU145. * *p* < 0.05, ** *p* < 0.01, *** *p* < 0.001, **** *p* < 0.0001.

Leveraging our findings on A3C expression, we selected 22RV1 and DU145 cells (low A3C expressers) for gain-of-function studies. We established stable A3C-overexpressing cell lines using overexpression plasmid. The experiment was divided into the Negative control (NC) group and the overexpression (OE) group, and the overexpression efficiency was confirmed through Western blot (Figure 4A) and was consistently high across three independent biological replicates (Figure 4B), providing robust models for subsequent functional assays.

We first assessed the impact of A3C on cell proliferation, and the CCK-8 assays over 72 h revealed that A3C overexpression significantly suppressed the proliferation rate of both 22RV1 and DU145 cells (Figure 4C). Furthermore, the anti-proliferative effect was further corroborated by a significant reduction in Ki67-positive cells (Figure 4D). We then evaluated the effect of A3C on metastatic potential. Wound healing assays demonstrated that A3C overexpression markedly impaired the migratory capacity of PCa cells, with inhibition rates at 24 and 48 h of 8% and 12% in 22RV1 cells and 12% and 13% in DU145 cells, respectively (Figure 4E,F). Furthermore, migration and invasion assays consistently showed that A3C overexpression led to a significant decrease in both the migratory and invasive abilities of the cells (Figure 4G,H).

### 3.5. Knockdown of A3C Promotes Proliferation, Migration, and Invasion of PCa Cells

**Figure 5:**
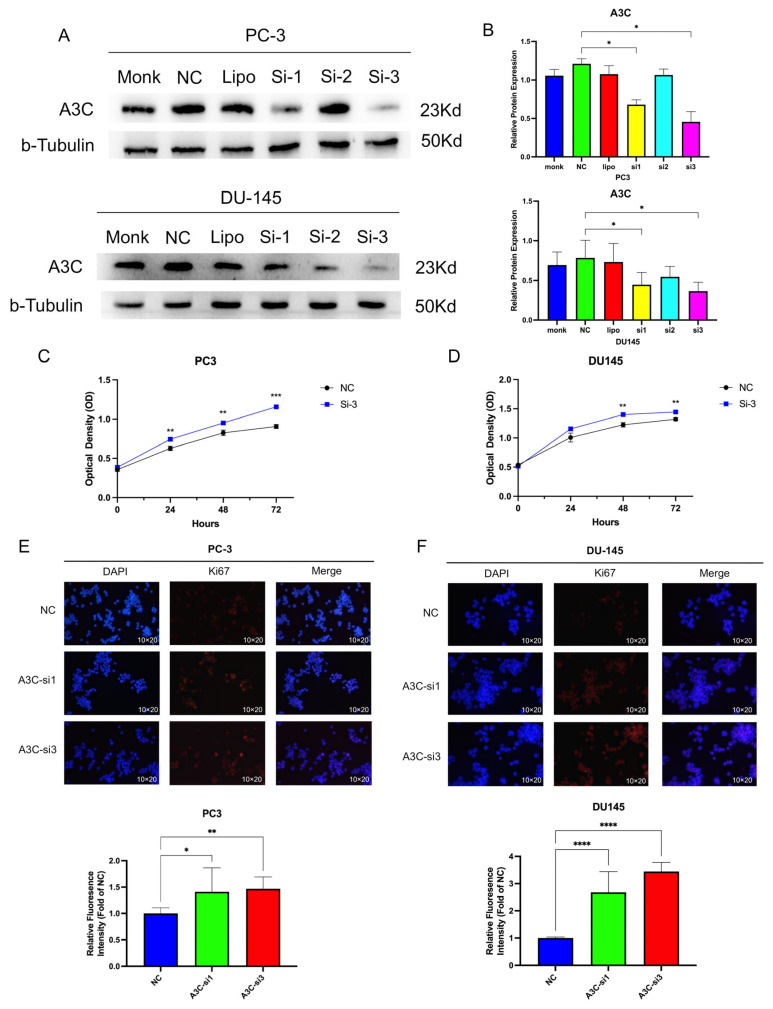
Downregulation of A3C promoted cell proliferation in PC-3 and DU145 cells. (**A**) Gel electrophoresis of knockdown. (**B**) Grayscale analysis for plots in (**A**). Data are presented as mean ± SD (*n* = 3 for PC-3 and *n* = 5 for DU145, independent experiments). One-way ANOVA followed by Dunnett’s multiple comparison test (show statistically meaningful *p* value only). (**C**,**D**) Results of CCK8 for PC-3 and DU145. Data are presented as mean ± SD (*n* = 4 independent experiments), two-way ANOVA followed by Šídák multiple comparison test. (**E**,**F**) Immunofluorescent pictures of Ki67 and relative fluorescence intensity (fold of NC) in PC-3 and DU145 cells, data are presented as mean ± SD (*n* = 3 independent experiments for each cell), one-way ANOVA followed by Dunnett’s multiple comparison test. * *p* <0.05, ** *p* <0.01, *** *p* <0.001, **** *p* <0.0001.

Based on the previous research results, we selected the PC3 and DU145 cell lines for knockdown experiments with small interfering RNA (siRNA). We used six groups to determine the knockdown efficiency through Western blotting: Monk (blank control), NC (negative control), Lipo (lipo2000 transfection reagent only), Si-1, Si-2, and Si-3 (Figure 5A,B). The results show that si3 had the highest knockdown efficiency, si1 had moderate efficiency, and si2 had poor efficiency. Thus, si1 and si3 were selected to construct A3C knockdown PCa cell models for subsequent experiments.

A 72-h CCK8 assay was performed using the si3 group. PC-3 and DU145 cells with A3C knockdown by si3 exhibited slightly enhanced proliferation (Figure 5C,D). Specifically, the 72-h cell count increased by 25% in si3-transfected PC-3 cells and 12% in si3-transfected DU145 cells relative to their respective controls. Additionally, Ki67 fluorescence staining revealed increased fluorescence intensity in the si1 and si3 groups, signifying a higher level of Ki67 expression as opposed to the control group (Figure 5E,F). These results suggest that A3C knockdown promotes PCa cell proliferation.

**Figure 6:**
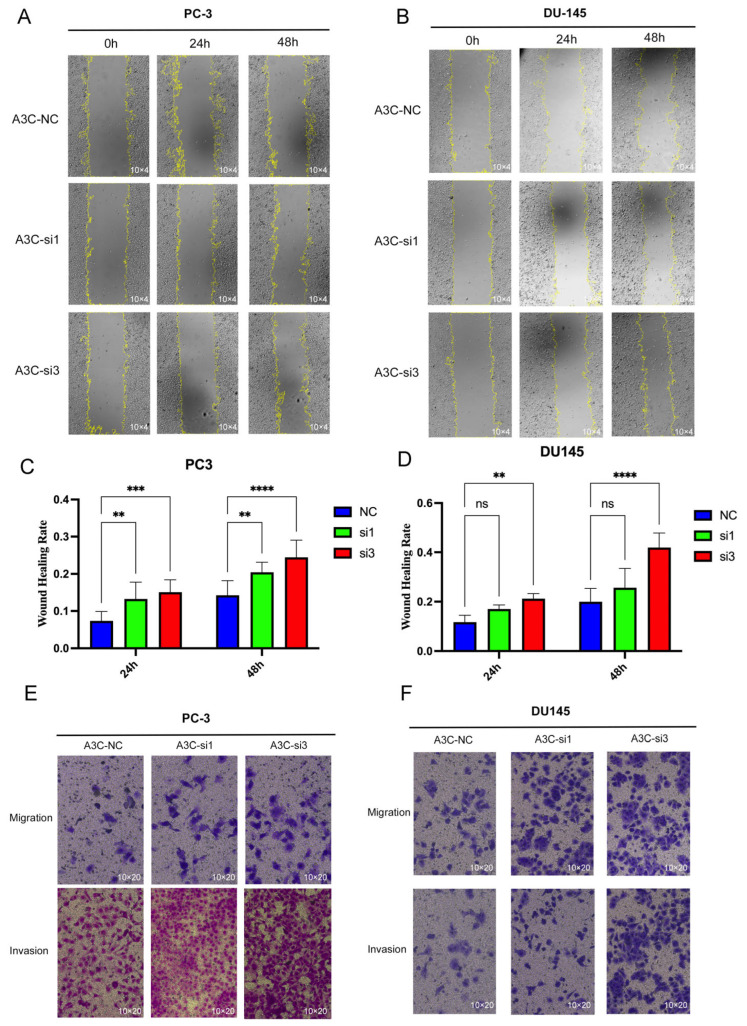
Downregulation of A3C promoted cell migration and invasion abilities in PC-3 and DU145 cells. (**A**–**D**) Wound healing assay in PC-3 and DU145 cells. Data are presented as mean ± SD (*n* = 3 independent experiments), two-way ANOVA followed by Šídák multiple comparison test. (**E**,**F**) Transwell assay in PC-3 and DU145 cells. ns, not significant, ** *p* < 0.01, *** *p* < 0.001, **** *p* < 0.0001.

Subsequently, wound healing results showed that, compared with the NC group, PCa cells with A3C knockdown by si3 exhibited increased migration capacity (Figure 6A,B), which was confirmed through three repeated experiments (Figure 6C,D); meanwhile, migration and invasion assays further demonstrated enhanced migration and invasion abilities in the knockdown groups (Figure 6E,F).

### 3.6. High A3C Expression Correlates with an Anti-Tumor Immune Microenvironment in PCa

To investigate the role of A3C in shaping the PCa immune microenvironment, we first evaluated its association with global immune features. Using the ESTIMATE algorithm, we found that high A3C expression was significantly correlated with Stromal, Immune, and ESTIMATE Scores (Supplementary Figure S2A), indicating a richer presence of both stromal and immune components. In contrast, a negative correlation was observed between A3C expression and tumor mutation burden (TMB) (Supplementary Figure S2B).

To delineate the impact of A3C on anti-tumor immunity, we systematically evaluated the relationship between its transcriptional levels and the immune cell. Comparative analysis revealed that tumors with high A3C expression exhibited significantly greater infiltration of anti-tumor effector cells, including CD8+ T cells, resting CD4+ memory T cells, and monocytes. Conversely, the immunosuppressive M2 macrophage population was enriched in the low-A3C group (Supplementary Figure S2C). This pattern was further confirmed through correlation analysis, which showed that A3C expression was positively correlated with resting CD4+ memory T cells and monocytes and, most notably, strongly negatively correlated with M2 macrophage infiltration (Supplementary Figure S2D,E).

Given the altered immune landscape, we explored its relationship with key immune checkpoints. While A3C showed only weak correlations with common inhibitory checkpoints like PD-1, PD-L1, and CTLA4 (Supplementary Figure S3A), it demonstrated a striking positive correlation with the costimulatory molecule CD40 (Supplementary Figure S3B). To functionally validate this bioinformatic prediction, we manipulated A3C expression in PCa cell lines and assessed CD40 protein levels through Western blot, and remarkably, A3C overexpression potently upregulated CD40 expression, whereas A3C knockdown suppressed it (Supplementary Figure S3C–E). This definitive experimental evidence positions A3C as a novel upstream regulator of CD40 in PCa cells.

### 3.7. Functional Enrichment Analysis and A3C Co-Expression Network Indicate Potential Downstream Mechanisms

We identified DEGs (2495 upregulated and 129 downregulated genes) between high- and low-A3C expression groups in the TCGA-PRAD cohort (FDR < 0.05, |log_2_FC| ≥ 1) (Supplementary Figure S4A). Functional enrichment analysis of these DEGs provided crucial insights into A3C’s function. GO terms were overwhelmingly enriched in immune-related processes, such as “lymphocyte-mediated immunity”, “T cell receptor complex”, and “antigen binding” (Supplementary Figure S4B). Consistent with this, a KEGG pathway analysis highlighted significant enrichment in key cancer–immune signaling pathways, including the PI3K-Akt, cAMP, and NOD-like receptor signaling pathways (Supplementary Figure S4C). Complementing these findings, single-sample GSEA (ssGSEA) further suggested a strong association between A3C activity and the JAK-STAT signaling pathway (Supplementary Figure S4D). Collectively, these bioinformatic predictions robustly position A3C as a central regulator of anti-tumor immunity and inflammatory signaling in the prostate cancer microenvironment. To pinpoint specific mechanistic partners of A3C, we identified 320 genes significantly co-expressed with A3C (R ≥ 0.7, *p* < 0.05) and constructed a protein–protein interaction (PPI) network. This analysis revealed that A3C is functionally networked with key regulators across three critical biological processes, suggesting a multifaceted mechanism for its tumor-suppressive role.

First, A3C expression was closely linked to the activation of the innate immune response. It showed significant co-expression with STING1 (also known as STING) and its downstream effector Caspase-1. Concordantly, the downstream inflammatory cytokines IL18 and IL1β were among the most significantly differentially expressed genes, strongly implicating A3C in the regulation of the STING-mediated inflammatory pathway (Supplementary Figure S5A).

Concurrently, our analysis suggested a role for A3C in maintaining genomic stability. We found a significant positive co-expression relationship between A3C and pivotal DNA damage protection genes, including GSTP1 and GPX3. This association implies that A3C may contribute to cellular defense against oxidative stress and DNA damage (Supplementary Figure S5B).

Finally, A3C was also significantly co-expressed with GAS1, a well-characterized growth arrest-specific protein and cell cycle regulator, indicating a potential additional role in modulating cell cycle progression (Supplementary Figure S5C).

The PPI network demonstrated robust functional interactions among these key players, indicating that they likely operate within interconnected biological modules rather than in isolation (Supplementary Figure S5D). Collectively, these co-expression analyses generate a compelling and unified mechanistic hypothesis: A3C may exert its tumor-suppressive effects by concurrently potentiating innate immune responses, enhancing DNA damage protection, and influencing cell cycle regulation.

### 3.8. A3C Upregulates Inflammatory Levels in PCa Cells

**Figure 7:**
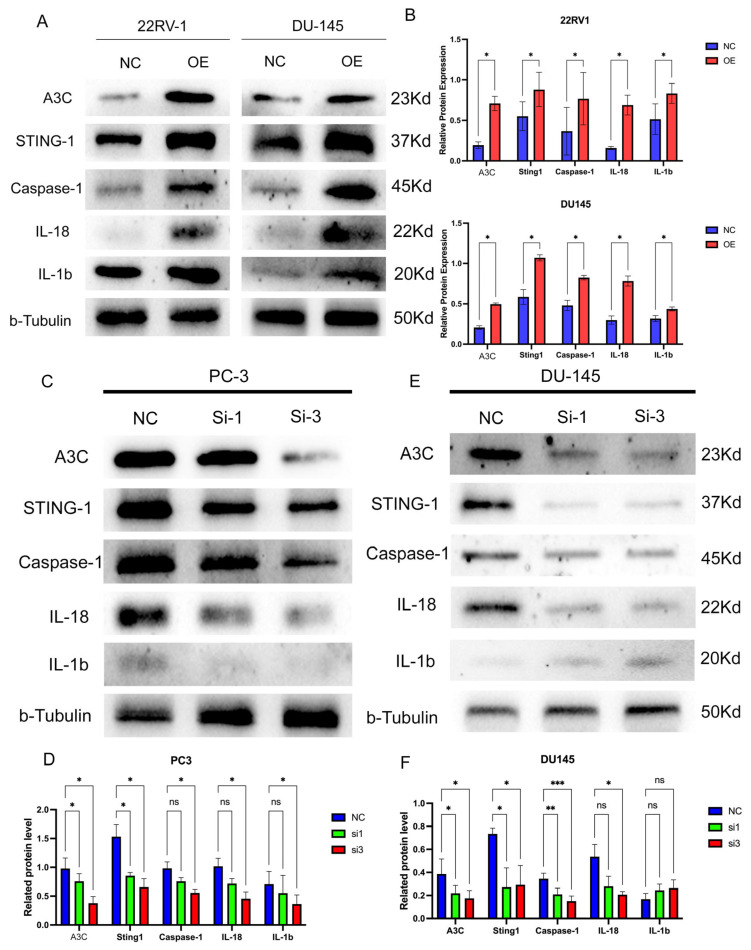
A3C upregulated the inflammation levels in PCa cells. (**A**) Overexpression of A3C upregulated the inflammation levels in 22RV1 and DU145 cells. (**B**) Grayscale analysis for plots in (**A**). Data are presented as mean ± SD (*n* = 3 independent experiments), two-tailed unpaired *t*-test. (**C**,**D**) Knockdown of A3C downregulated the inflammation levels in PC-3 cells and its related grayscale analysis, shown as mean ± SD (*n* = 3 independent experiments); one-way ANOVA followed by Dunnett’s multiple comparison test. (**E**,**F**) Knockdown of A3C downregulated the inflammation levels in DU145 cells, and its related grayscale analysis, shown as mean ± SD (*n* = 3 independent experiments); one-way ANOVA followed by Dunnett’s multiple comparison test. ns, not significant, * *p* < 0.05, ** *p* < 0.01, *** *p* < 0.001.

First, the effect of A3C on inflammatory levels was investigated in A3C-overexpressing PCa cell lines (22RV1 and DU145). The results showed that the overexpression of A3C significantly increased the expression levels of STING1 and Caspase1 in PCa cells; concurrently, the expression levels of downstream inflammatory factors (IL18 and IL1β) were also elevated (Figure 7A,B).

Furthermore, to confirm the role of A3C in regulating inflammatory levels, reverse validation was performed in A3C knockdown PCa cell lines (PC-3 and DU145). The results demonstrated that A3C knockdown significantly decreased the expression of STING1 and Caspase1, accompanied by reduced expression of downstream inflammatory factors (IL18 and IL1β) (Figure 7C–F).

### 3.9. A3C Upregulates the Expression of DNA Damage Protection-Related Proteins

**Figure 8:**
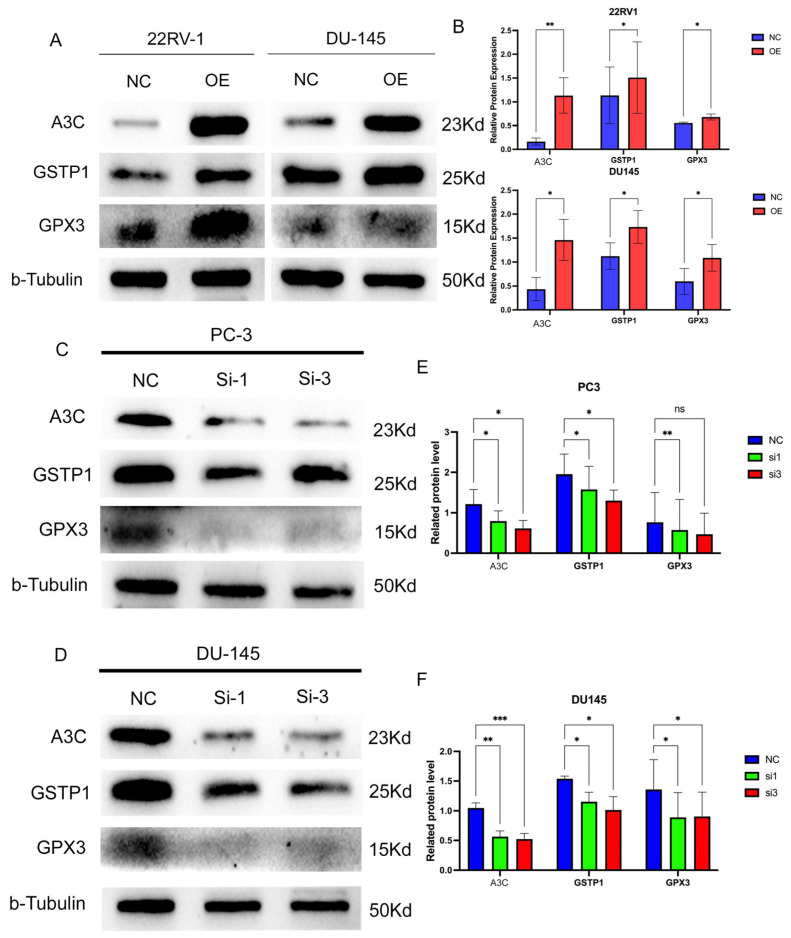
A3C upregulated the expressions of DNA damage protection-related proteins in PCa cells. (**A**) Overexpression of A3C upregulated the expressions of GSTP1 and GPX3 in 22RV1 and DU145 cells. (**B**) Grayscale analysis for plots in (**A**). Data are presented as mean ± SD (*n* = 3 independent experiments), two-tailed unpaired *t*-test. (**C**,**D**) Knockdown of A3C downregulated the expressions of GSTP1 and GPX3 in PC-3 cells and DU145 cells. (**E**,**F**) Grayscale analysis for (**C**,**D**). Data are presented by mean ± SD (*n* = 3 independent experiments each cell line); one-way ANOVA followed by Dunnett’s multiple comparison test. ns, not significant, * *p* < 0.05, ** *p* < 0.01, *** *p* < 0.001.

Subsequently, we investigated the effect of A3C on DNA damage protection-related proteins (GSTP1 and GPX3). In A3C-overexpressing 22RV1 and DU145 cells, the expression levels of GSTP1 and GPX3 were found to be increased (Figure 8A,B). Conversely, after A3C knockdown, the levels of GSTP1 and GPX3 decreased accordingly (Figure 8C–F). The results indicate that A3C upregulates the expression levels of DNA damage protection-related proteins such as GSTP1 and GPX3.

### 3.10. A3C Has the Potential to Inhibit the Cell Cycle by Upregulating GAS1 Protein Expression

**Figure 9:**
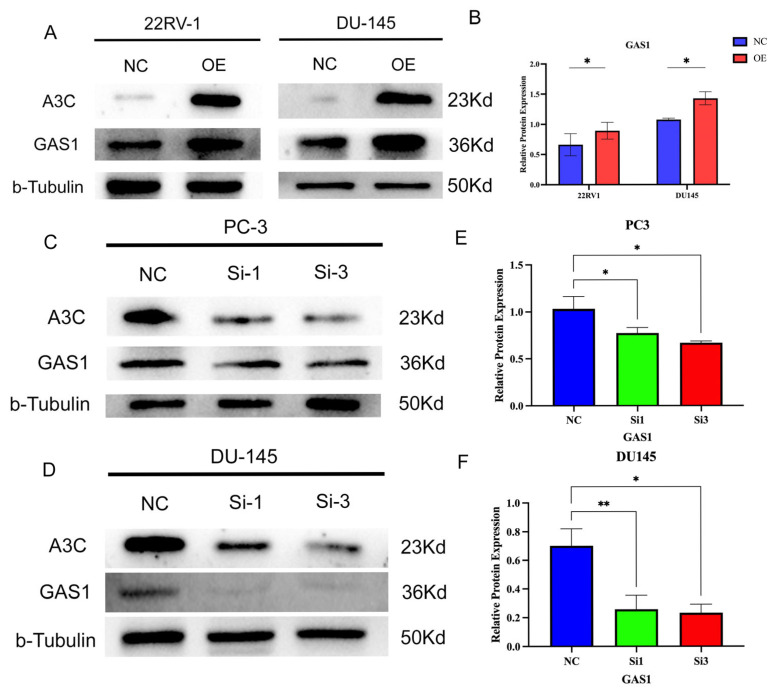
A3C blocked the cell cycle of PCa cells by upregulating the expression of GAS1. (**A**) Overexpression of A3C upregulated the expressions of GAS1 in 22RV1 and DU145 cells. (**B**) Grayscale analysis for plots in (**A**). Data are presented as mean ± SD (*n* = 3 independent experiments), two-tailed unpaired *t*-test. (**C**,**D**) Knockdown of A3C downregulated the expressions of GAS1 in PC-3 cells and DU145 cells. (**E**,**F**) Grayscale analysis for (**C**,**D**). Data are presented as mean ± SD (*n* = 3 independent experiments each cell line), one-way ANOVA followed by Dunnett’s multiple comparison test. * *p* < 0.05, ** *p* < 0.01.

Finally, we investigated the effect of A3C expression on GAS1 protein expression. Western blot experiments confirmed that A3C overexpression upregulated GAS1 expression levels (Figure 9A,B), whereas A3C knockdown downregulated GAS1 levels. These findings were verified through more than three repeated experiments (Figure 9C–F). The above results indicate that A3C has the potential to inhibit the cell cycle of PCa cells by upregulating GAS1.

## 4. Discussion

In this study, we combined WGCNA, differential expression gene analysis, and LASSO regression to screen two PCa-related genes (*A3C* and *AOX1*) from TCGA and GEO PCa datasets. Studies have revealed that a diminished methylation status of the *AOX1* gene correlates with a decreased rate of biochemical recurrence and more favorable prognostic outcomes in PCa patients, while further studies have demonstrated that hypermethylation—an epigenetic modification that suppresses *AOX1* expression—enhances the migratory and invasive potential of prostate cancer cells, collectively providing compelling evidence that *AOX1* functions as a tumor suppressor in PCa [16,17]. Given the well-established research on *AOX1* in PCa, we focused on *A3C* for subsequent investigations. Previous studies have only proposed a decreased expression level of *A3C* in PCa patients and its potential prognostic value, but research on its specific mechanism is still lacking [14,15].

Integrating bioinformatics analysis, IHC, and cellular experiments, we found that *A3C* expression was significantly downregulated in PCa. Consistent findings have also been reported, which demonstrated that the expression level of *A3C* in pathological specimens of PCa is significantly lower than that in the benign control group [15]. Patients with high *A3C* expression exhibited better PFS and DSS, along with lower Gleason scores and T stages. Consistent with this, a previous study reported that *A3C* expression was lower in high-grade PCa than in low-grade tumors [13], collectively confirming that *A3C* correlates with favorable prognosis in PCa.

We further explored the association between *A3C* and the PCa immune microenvironment. High *A3C* expression was linked to higher immune scores (including Stromal Score, Immune Score, and ESTIMATE Score). Notably, elevated immune scores are associated with better prognosis and can predict immune therapeutic efficacy [18], suggesting that *A3C* may have potential in predicting immune treatment outcomes for PCa. Additionally, high *A3C* expression correlated with increased CD8+ T cell infiltration—CD8+ T cells are key mediators of anti-tumor immunity, capable of inducing tumor cell programmed death by releasing perforin, tumor necrosis factor, and interferon [19,20]. In contrast, *A3C* expression was significantly negatively correlated with M2 macrophage infiltration; M2 macrophages are associated with poor PCa prognosis and promote tumor progression by secreting cytokines that reshape the immune microenvironment [21]. Furthermore, *A3C* showed weak correlations with immune checkpoints, including *PDCD1*, *CD274*, and *CTLA4*, and a significant positive correlation with the immune checkpoint *CD40*. Western blot experiments confirmed that *A3C* positively regulates *CD40* expression. *CD40*, a member of the tumor necrosis factor superfamily, induces growth arrest and apoptosis when overexpressed in tumor cells [22] and activates dendritic cells to enhance CD4+ and CD8+ T cell anti-tumor effects [23]. A phase I clinical trial also showed that *CD40* activation enhanced anti-tumor immunity in patients with castration-resistant PCa [24], further validating *A3C*’s tumor-suppressive role and its potential as a target for *CD40*-related immunotherapy. These findings indicate that *A3C* remodels the PCa immune microenvironment, with high *A3C* expression correlating with enhanced immune function.

Cellular experiments involving *A3C* expression manipulation (overexpression or knockdown) in multiple PCa cell lines, combined with CCK8, Ki67 fluorescence staining, wound healing, and Transwell assays, demonstrated that *A3C* overexpression inhibited cell proliferation, migration, and invasion, while *A3C* knockdown partially promoted these malignant phenotypes—confirming *A3C*’s tumor-suppressive role. Existing studies have only partially indicated the differential expression of *A3C* in PCa and its potential value as a biomarker [14,15]. Thus, this study represents the first investigation to elucidate the inhibitory mechanisms of PCa mediated by *A3C*, though *A3C*’s tumor-suppressive potential has been suggested in other cancers: in gastric cancer, CagA overexpression downregulated both *A3C* and the tumor suppressor PTEN [25]; in breast cancer, the tumor-suppressive *lncRNA GAS5* and its target *A3C* were both downregulated in tumor tissues [26].

Functional enrichment analysis revealed that *A3C*-related differentially expressed genes were enriched in the Jak-Stat pathway (enhanced Jak-Stat signaling is associated with PCa drug resistance [27]), PI3K-AKT pathway (activation promotes PCa cell growth, angiogenesis, and metastasis [28]), and immune-related pathways such as the NOD-like receptor pathway, supporting *A3C* as an immune-related gene in PCa.

Based on co-expression network and protein–protein interaction analyses, *A3C* may exert tumor-suppressive effects through three mechanisms: First, it upregulates STING1 (a key protein in the cGAS-Sting pathway that regulates inflammation and immunity [29,30,31,32]) and modulates Caspase1 (involved in inflammasome activation and pyroptosis [33,34]) and its downstream inflammatory factors IL18 and IL1β (which recruit immune cells [35]). Since Sting pathway activation affects inflammasome-related protein expression [36], *A3C* may enhance cellular inflammatory levels by upregulating STING1. Second, A3C upregulates *GSTP1* and *GPX3*: *GSTP1* has antioxidant and DNA-protective effects, and high *GSTP1* expression inhibits PCa cell proliferation [37,38,39]. *GPX3* scavenges reactive oxygen species to protect DNA, with its overexpression inhibiting PCa cell growth and metastasis and low expression correlating with poor prognosis [40,41]. Third, *A3C* upregulates *GAS1*, a protein that arrests the cell cycle at the S phase. In PCa, high *GAS1* expression inhibits proliferation and is downregulated in high-grade tumors [42,43,44], suggesting that *A3C* may block the cell cycle via *GAS1* upregulation.

It should be noted that the above speculations about A3C’s tumor-suppressive mechanisms are all derived from the bioinformatics analyses and cellular experiments in this study. Further verification of their reliability and specificity will necessitate targeted follow-up studies, including rescue experiments and molecular mechanism validation assays. Moreover, the quantitative analysis of partial Western blot data was subject to technical limitations. Therefore, future studies are warranted to validate these findings under optimized experimental conditions or using in vivo models.

## 5. Conclusions

*A3C* is a prognosis-related gene in PCa associated with favorable clinical outcomes; it correlates with higher immune scores and increased CD8+ T cell infiltration, positively regulates the expression of immune checkpoint *CD40*, and plays a role in remodeling the immune microenvironment. Additionally, *A3C* may inhibit the malignant behaviors of PCa cells through regulating key molecules involved in synergistic mechanisms: upregulating *STING1* and its downstream molecules in intracellular inflammation, increasing the expression of DNA damage protection-related proteins such as *GSTP1* and *GPX3*, and upregulating *GAS1* to suppress the cell cycle.

## Data Availability

The data generated in this study are publicly accessible via the GEO and TCGA databases, specifically for PCa-related datasets. Additionally, the IHC data for prostate cancer and normal prostate tissues utilized in this research were retrieved from The Human Protein Atlas database.

## Supplementary Materials

The following supporting information can be downloaded at https://www.mdpi.com/article/10.3390/cancers18010170/s1. Figure S1: A3C and AOX1 could diagnose PCa well. Figure S2: High expression of A3C is correlated with better immunity in the tumor microenvironment in PCa. Figure S3: A3C regulates the expression of CD40; Figure S4: A3C is involved in tumor immune-related functions and pathways in PCa. Figure S5: Co-expressed gene network of A3C indicated three possible mechanisms in tumor suppressing. Figure S6: Original images for blots.

## Abbreviations

A3s	APOBEC3/Apolipoprotein B mRNA-Editing Enzyme Catalytic Polypeptide-Like 3
PCa	Prostate Cancer
DSS	Disease-Specific Survival
KEGG	Kyoto Encyclopedia of Genes and Genomes
LASSO	Least Absolute Shrinkage and Selection Operator
AUC	Area Under Curve
CCK8	Cell Counting Kit-8
ROC	Receiver Operating Characteristic
One-way ANOVA	One-Way Analysis of Variance
PFS	Progression-Free Survival
ssGSEA	Single-Sample Gene Set Enrichment
GEO	Gene Expression Omnibus
TMB	Tumor Mutation Burden
WGCNA	Weighted Gene Correlation Network Analysis
