# Supplementary material for: APOBEC3C Suppresses Prostate Cancer by Regulating Key Molecules Involved in Cellular Inflammation, Cell Cycle Arrest, and DNA Damage Response
Source: Cancers (Basel). 2026 Jan 3;18(1):170. doi: 10.3390/cancers18010170 (PMC12785094; doi:10.3390/cancers18010170)
Supplement: Supplementary file 1 [file cancers-18-00170-s001.zip › Supplementary Figure S6.pdf]

Fig.2. F

WPMY-1	+	-	-	-
22RV1	-	+	-	-
Du145	-	-	+	-
Pc-3	-	-	-	+

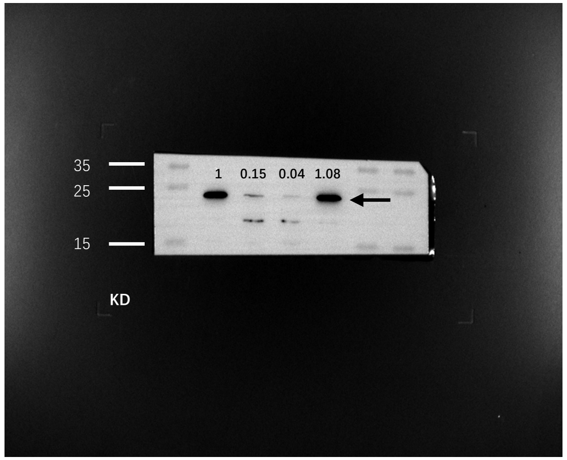

APOBEC3C (23kDa)

WPMY-1	+	-	-	-
22RV1	-	+	-	-
Du145	-	-	+	-
Pc-3	-	-	-	+

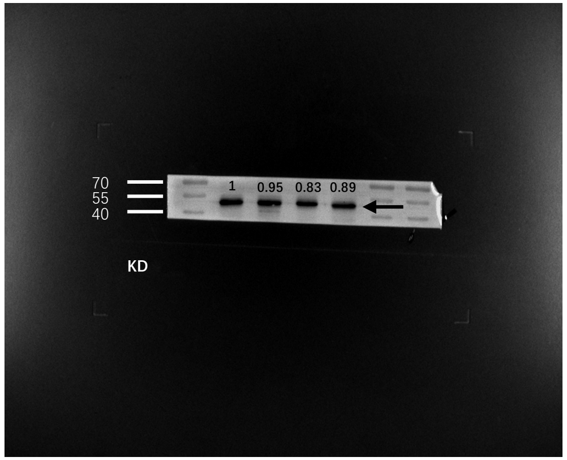

b-Tubulin (50kDa)

WPMY-1	+	-	-	-
22RV1	-	+	-	-
Du145	-	-	+	-
Pc-3	-	-	-	+

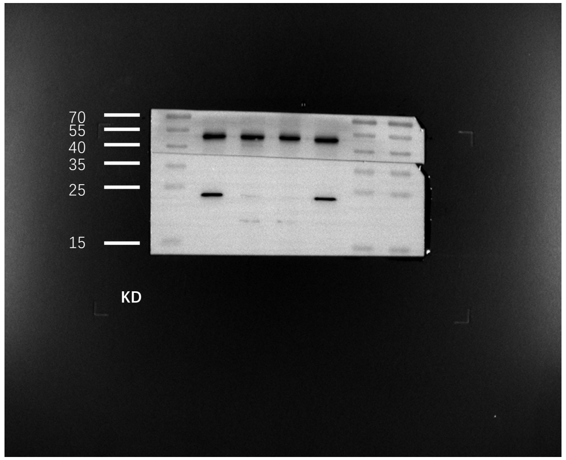

Merged

Fig.4. A

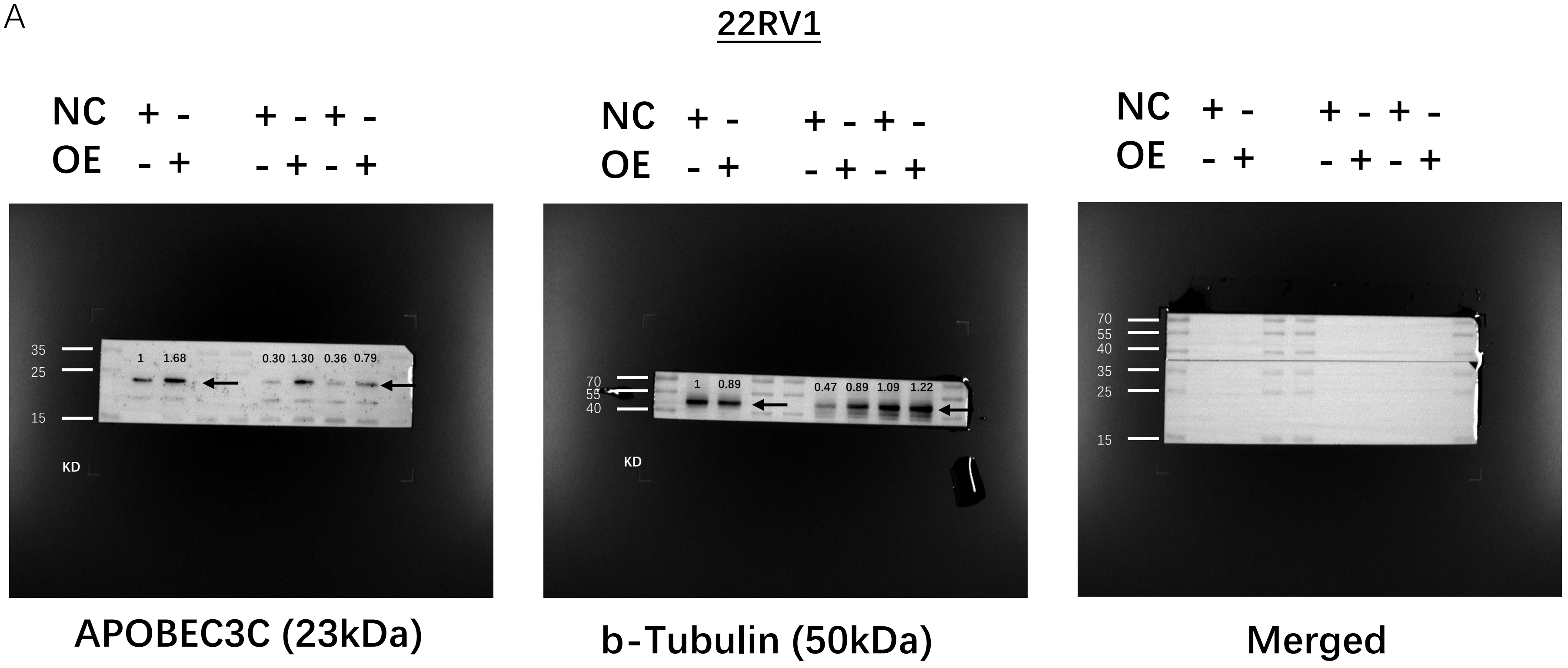
22RV1
NC + - + - + -
OE - + - + - +
35
25
15
KD
1 1.68 0.30 1.30 0.36 0.79
APOBEC3C (23kDa)
NC + - + - + -
OE - + - + - +
70
55
40
KD
1 0.89 0.47 0.89 1.09 1.22
b-Tubulin (50kDa)
NC + - + - + -
OE - + - + - +
70
55
40
35
25
15
Merged

Fig.4. A

DU-145

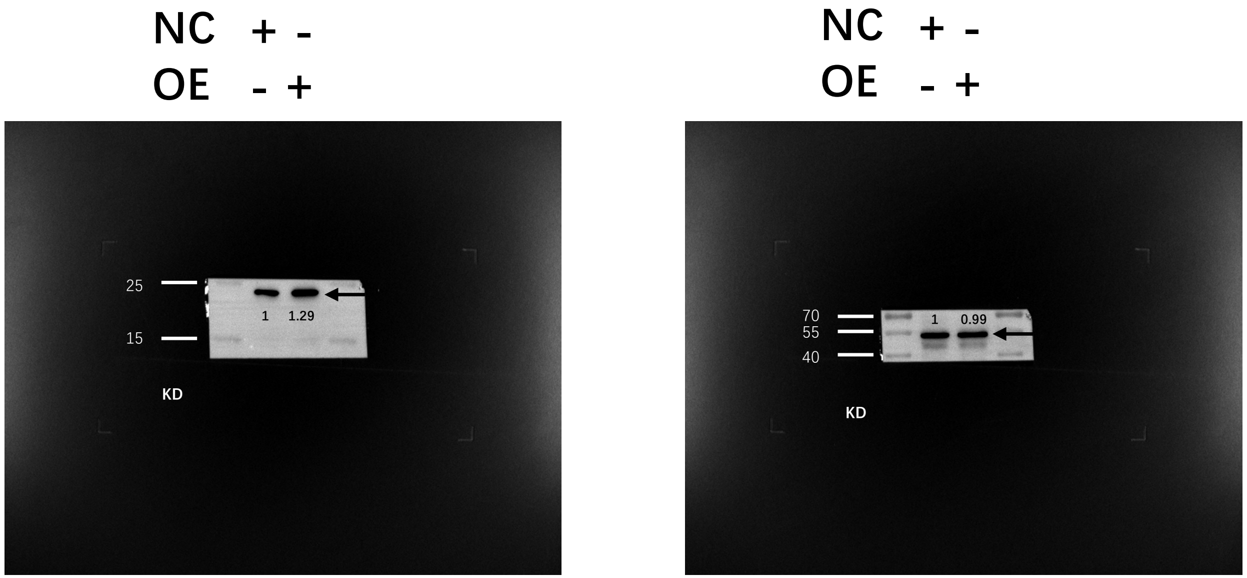

APOBEC3C (23kDa)

b-Tubulin (50kDa)

Fig.5. A

<u>**PC-3**</u>

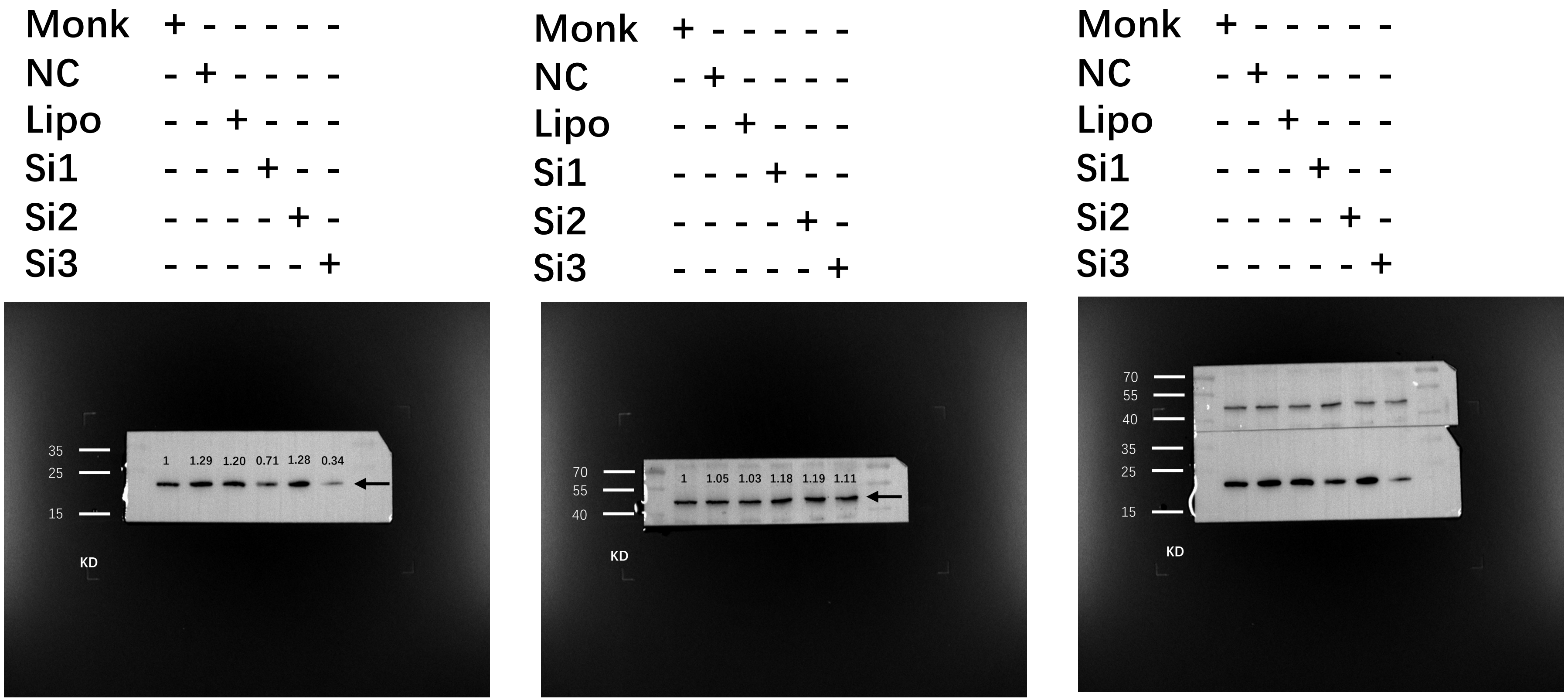

APOBEC3C (23kDa)   b-Tubulin (50kDa)   Merged

Fig.5. A

Du-145

Monk	+	-	-	-	-	-
NC	-	+	-	-	-	-
Lipo	-	-	+	-	-	-
Si1	-	-	-	+	-	-
Si2	-	-	-	-	+	-
Si3	-	-	-	-	-	+

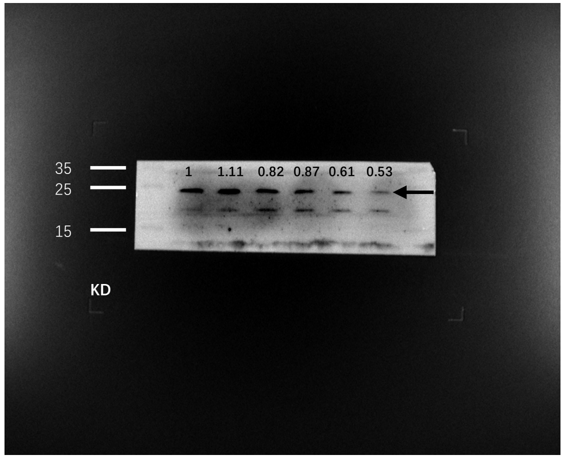

APOBEC3C (23kDa)

Monk	+	-	-	-	-	-
NC	-	+	-	-	-	-
Lipo	-	-	+	-	-	-
Si1	-	-	-	+	-	-
Si2	-	-	-	-	+	-
Si3	-	-	-	-	-	+

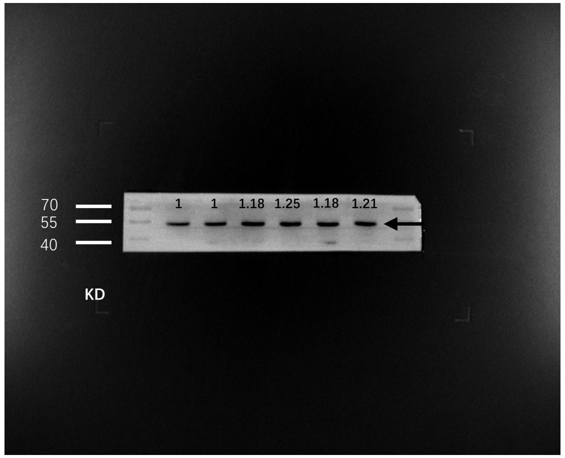

b-Tubulin (50kDa)

Fig.7. A

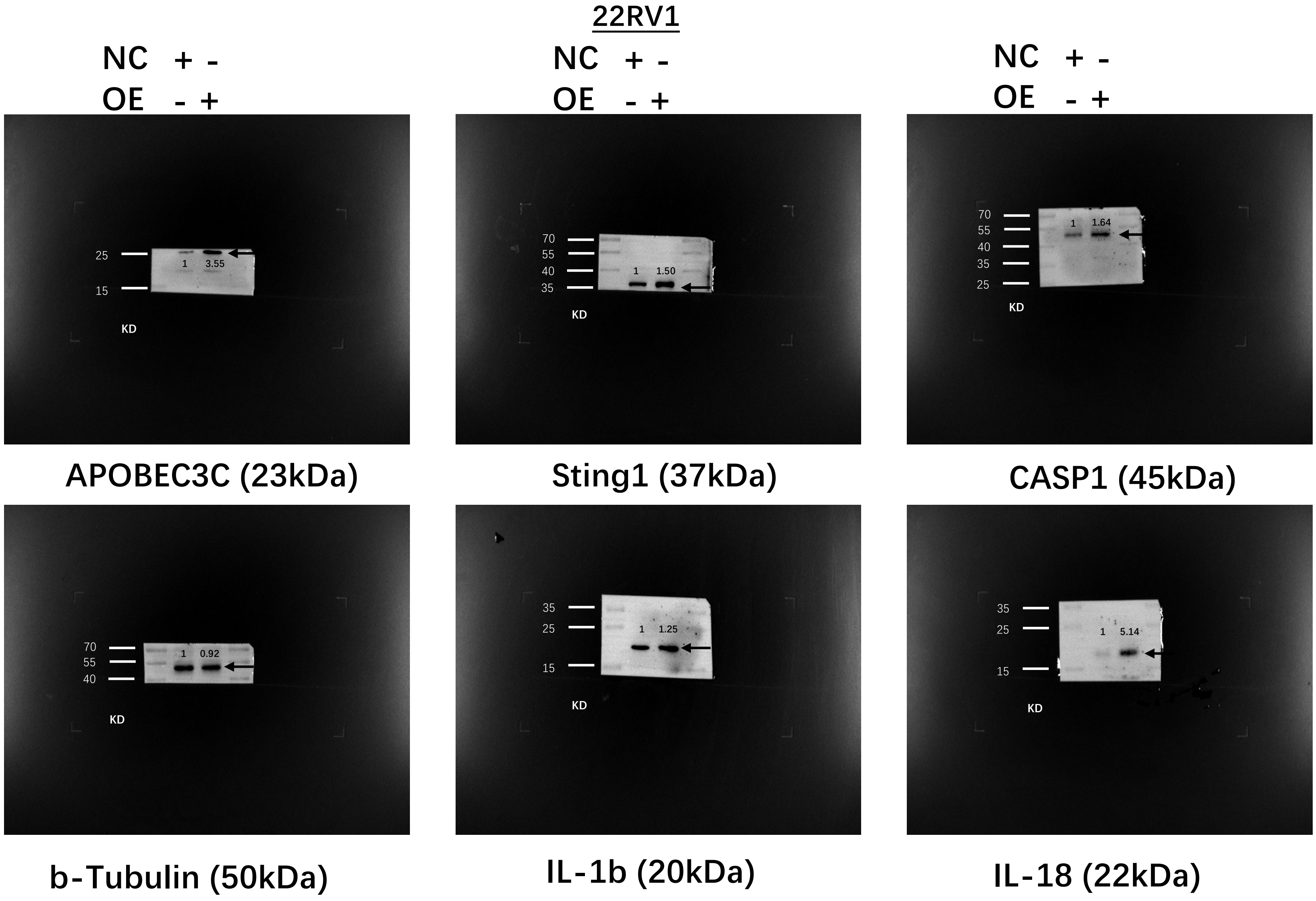
22RV1
NC + -
OE - +
25
15
KD
1
3.55
APOBEC3C (23kDa)
70
55
40
35
KD
1
1.50
Sting1 (37kDa)
70
55
40
35
25
KD
1
1.64
CASP1 (45kDa)
70
55
40
KD
1
0.92
b-Tubulin (50kDa)
35
25
15
KD
1
1.25
IL-1b (20kDa)
35
25
15
KD
1
5.14
IL-18 (22kDa)

Fig.7. A

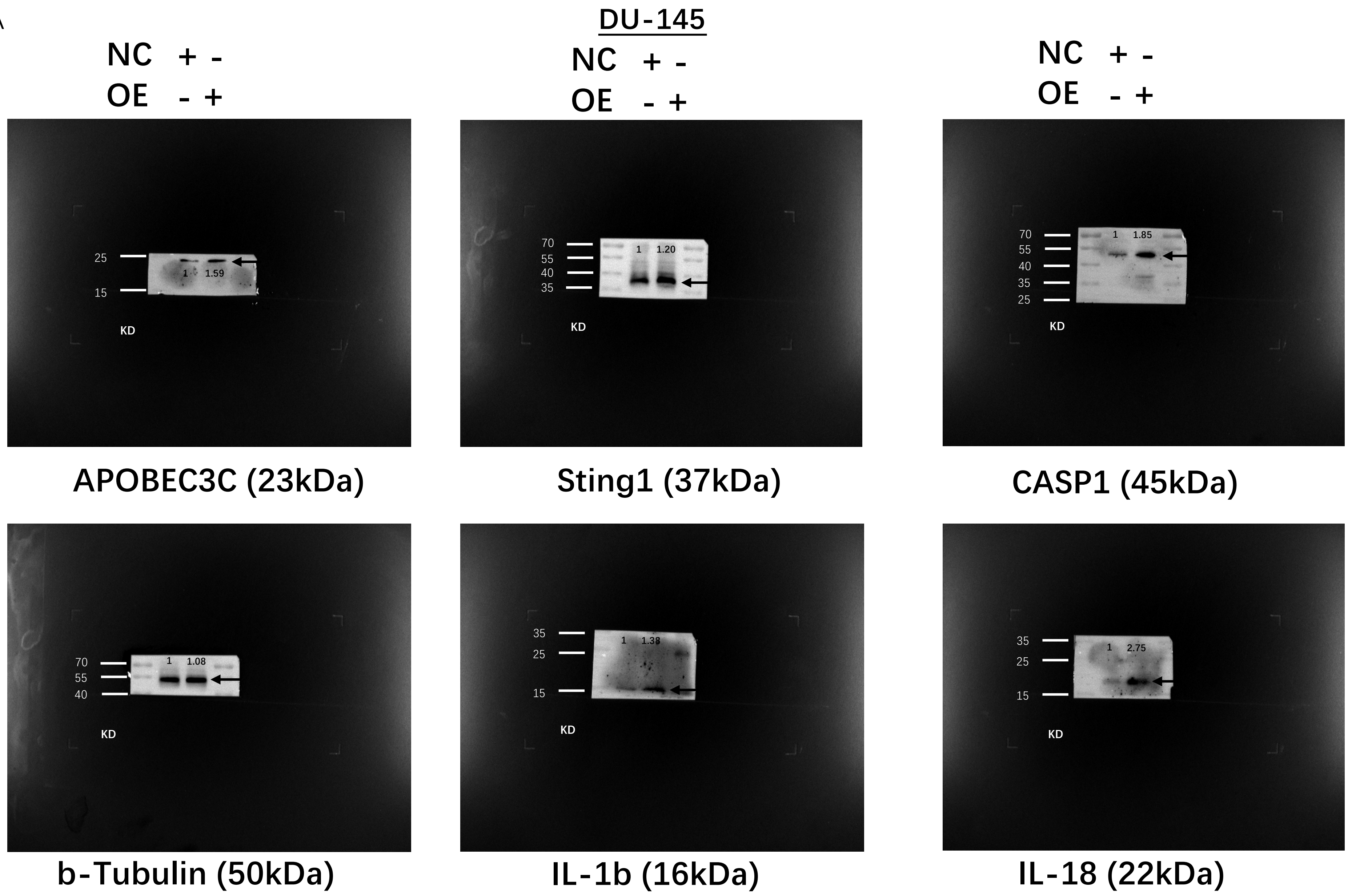
DU-145
NC + -
OE - +
NC + -
OE - +
NC + -
OE - +
25
15
KD
1
1.59
APOBEC3C (23kDa)
70
55
40
35
KD
1
1.20
Sting1 (37kDa)
70
55
40
35
25
KD
1
1.85
CASP1 (45kDa)
70
55
40
KD
1
1.08
b-Tubulin (50kDa)
35
25
15
KD
1
IL-1b (16kDa)
35
25
15
KD
1
2.75
IL-18 (22kDa)

Fig.7. C

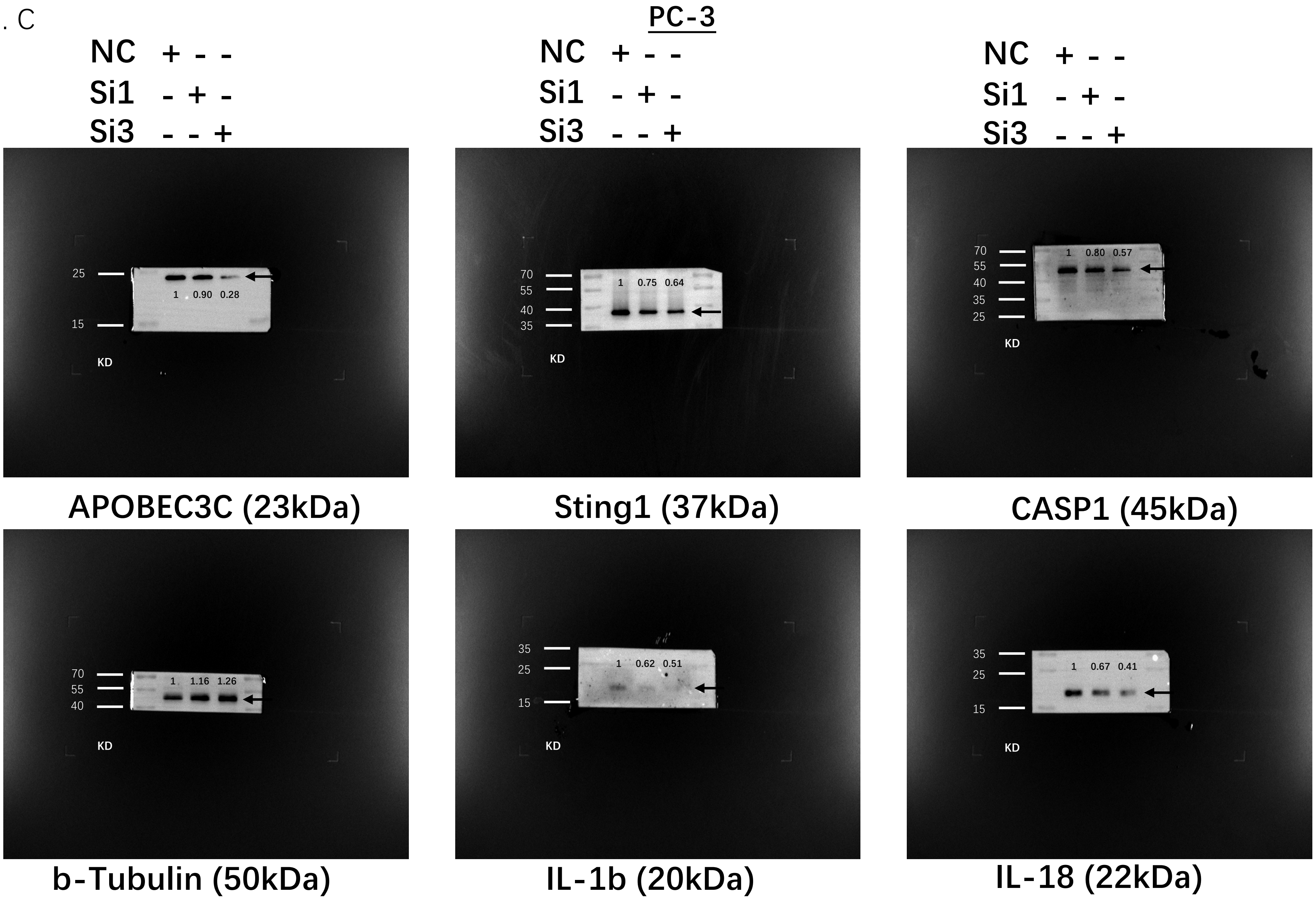

PC-3
NC + - -
Si1 - + -
Si3 - - +
25
15
KD
1 0.90 0.28
APOBEC3C (23kDa)
70
55
40
35
KD
1 0.75 0.64
Sting1 (37kDa)
70
55
40
35
25
KD
1 0.80 0.57
CASP1 (45kDa)
70
55
40
KD
1 1.16 1.26
b-Tubulin (50kDa)
35
25
15
KD
1 0.62 0.51
IL-1b (20kDa)
35
25
15
KD
1 0.67 0.41
IL-18 (22kDa)

Fig.7. E

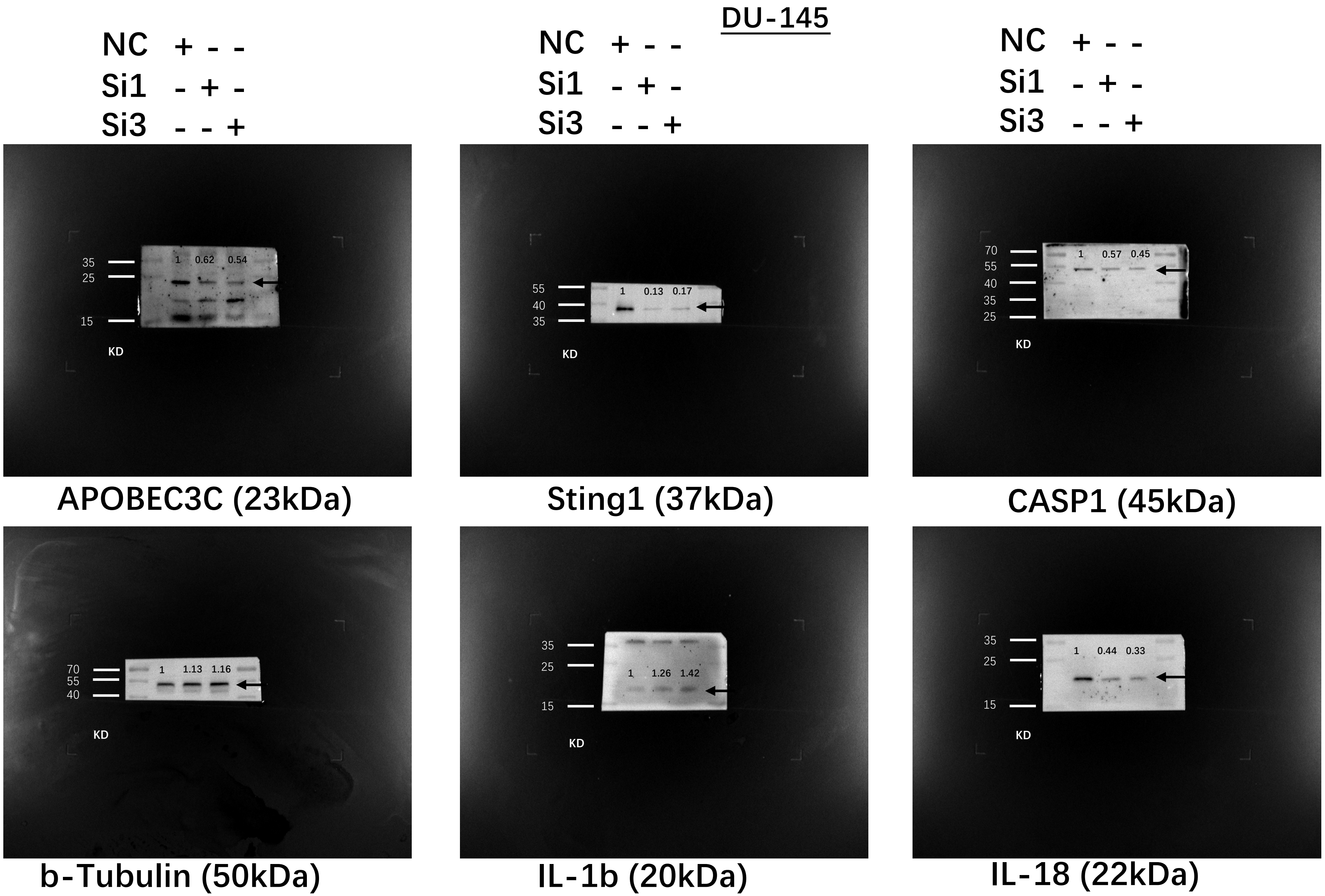

DU-145
NC + - -
Si1 - + -
Si3 - - +
35
25
15
KD
1 0.62 0.54
APOBEC3C (23kDa)
55
40
35
KD
1 0.13 0.17
Sting1 (37kDa)
70
55
40
35
25
KD
1 0.57 0.45
CASP1 (45kDa)
70
55
40
KD
1 1.13 1.16
b-Tubulin (50kDa)
35
25
15
KD
1 1.26 1.42
IL-1b (20kDa)
35
25
15
KD
1 0.44 0.33
IL-18 (22kDa)

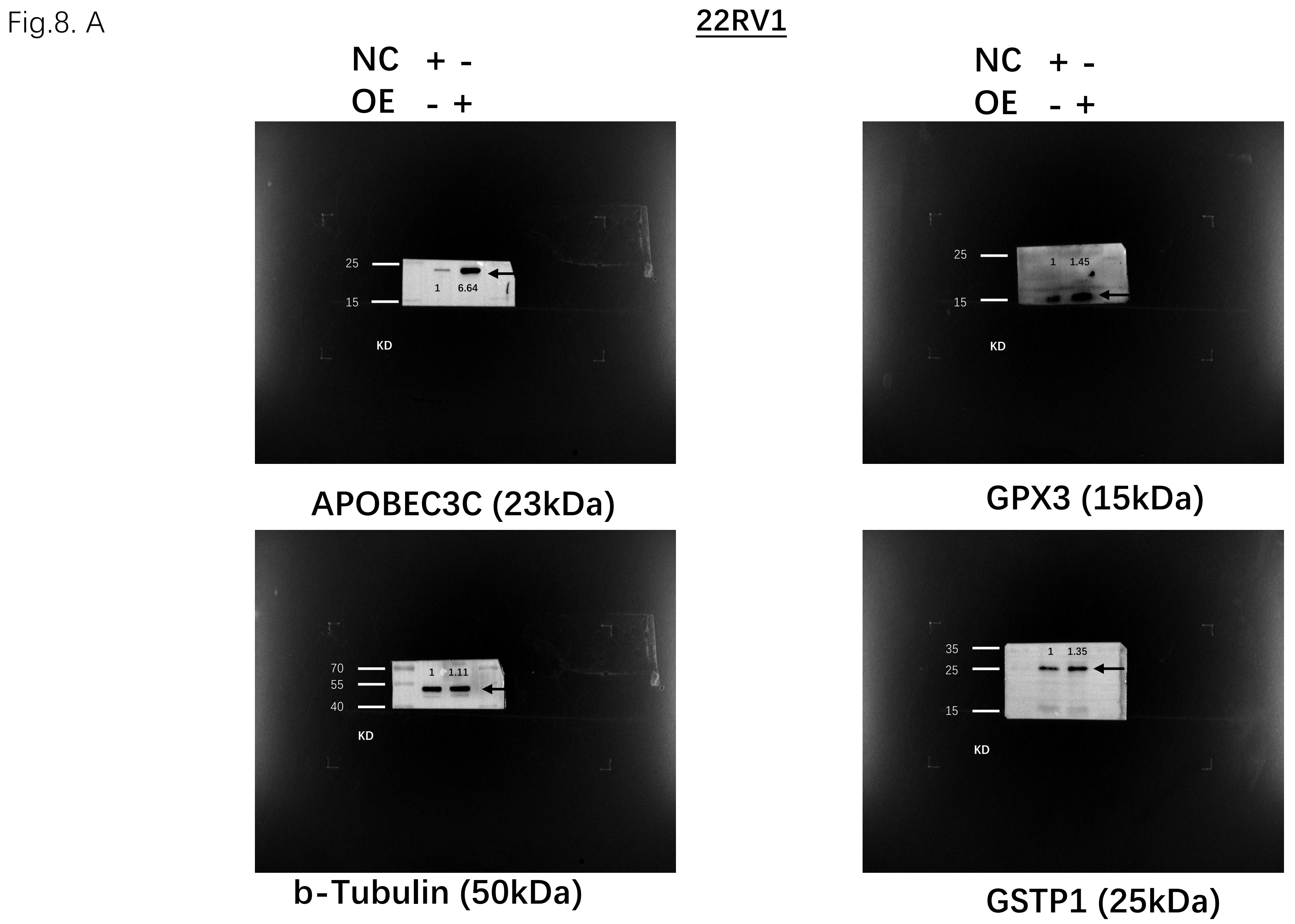
Fig.8. A
22RV1
NC + -
OE - +
25
15
KD
1
6.64
APOBEC3C (23kDa)
NC + -
OE - +
25
15
KD
1
1.45
GPX3 (15kDa)
70
55
40
KD
1
1.11
b-Tubulin (50kDa)
35
25
15
KD
1
1.35
GSTP1 (25kDa)

Fig.8. A

DU-145

NC + -   NC + -

OE - +   OE - +

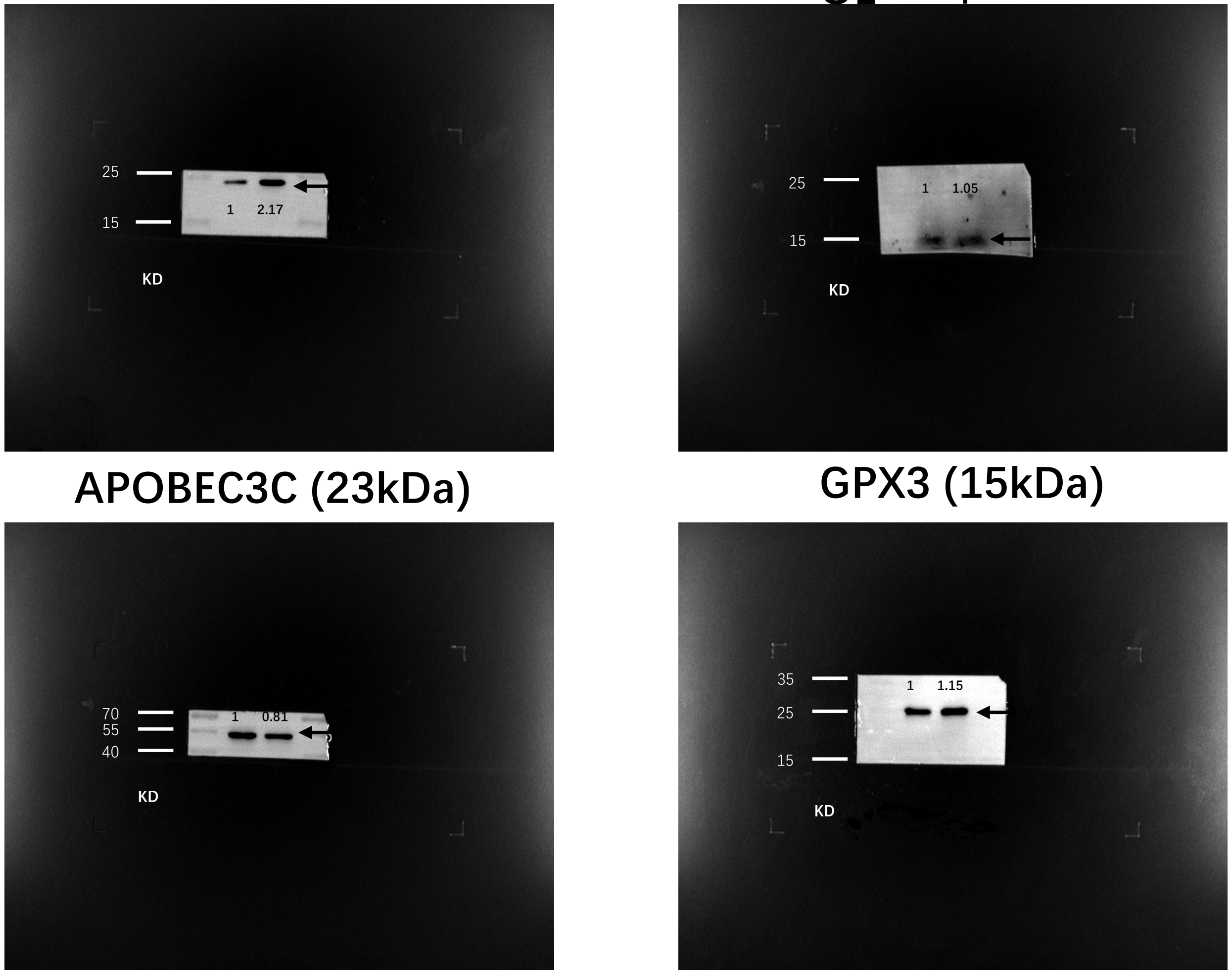

APOBEC3C (23kDa)   GPX3 (15kDa)

b-Tubulin (50kDa)   GSTP1 (25kDa)

Fig.8. C

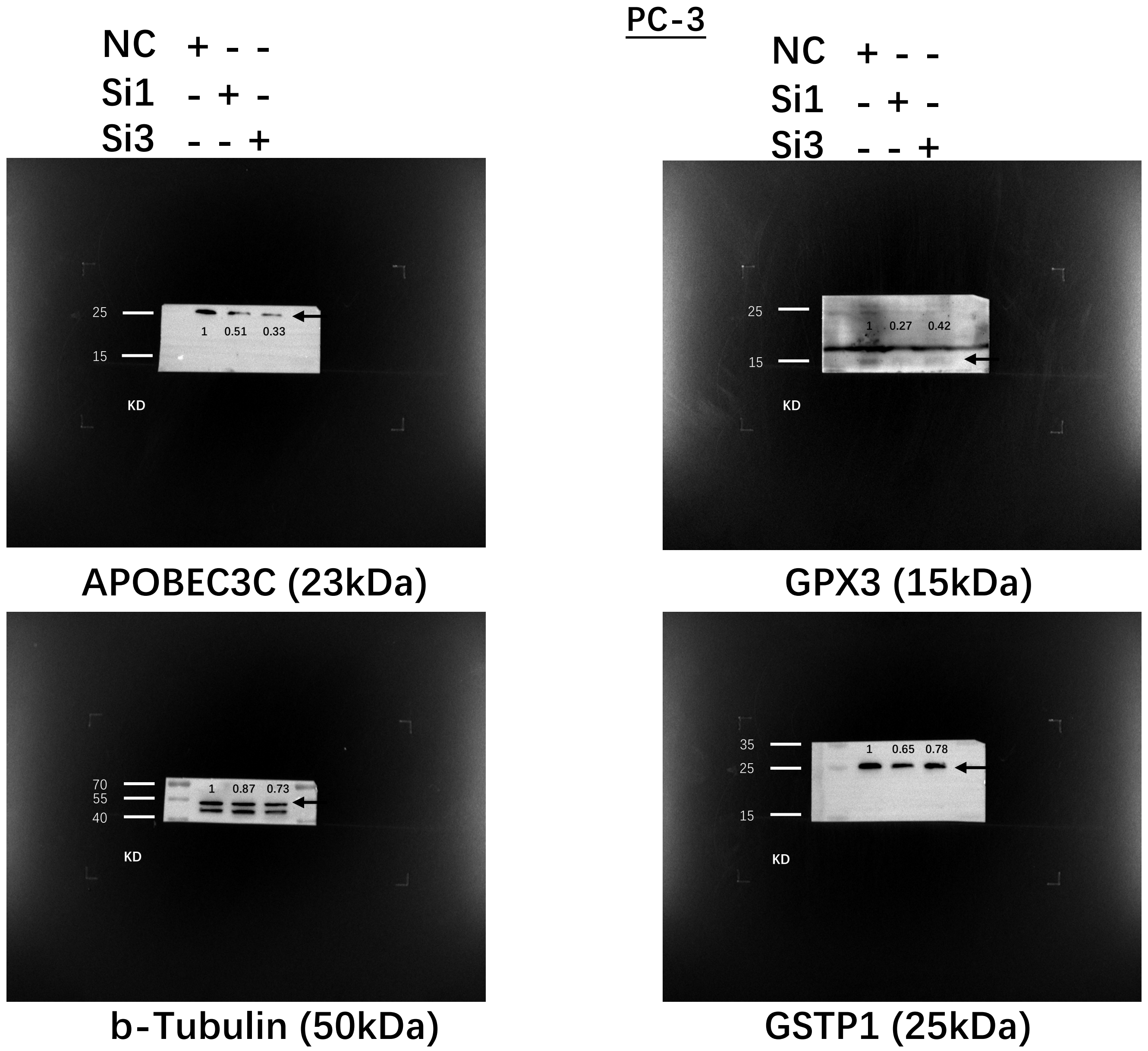

PC-3
NC + - -
Si1 - + -
Si3 - - +
25
15
KD
1 0.51 0.33
APOBEC3C (23kDa)
25
15
KD
1 0.27 0.42
GPX3 (15kDa)
70
55
40
KD
1 0.87 0.73
b-Tubulin (50kDa)
35
25
15
KD
1 0.65 0.78
GSTP1 (25kDa)

Fig.8. E

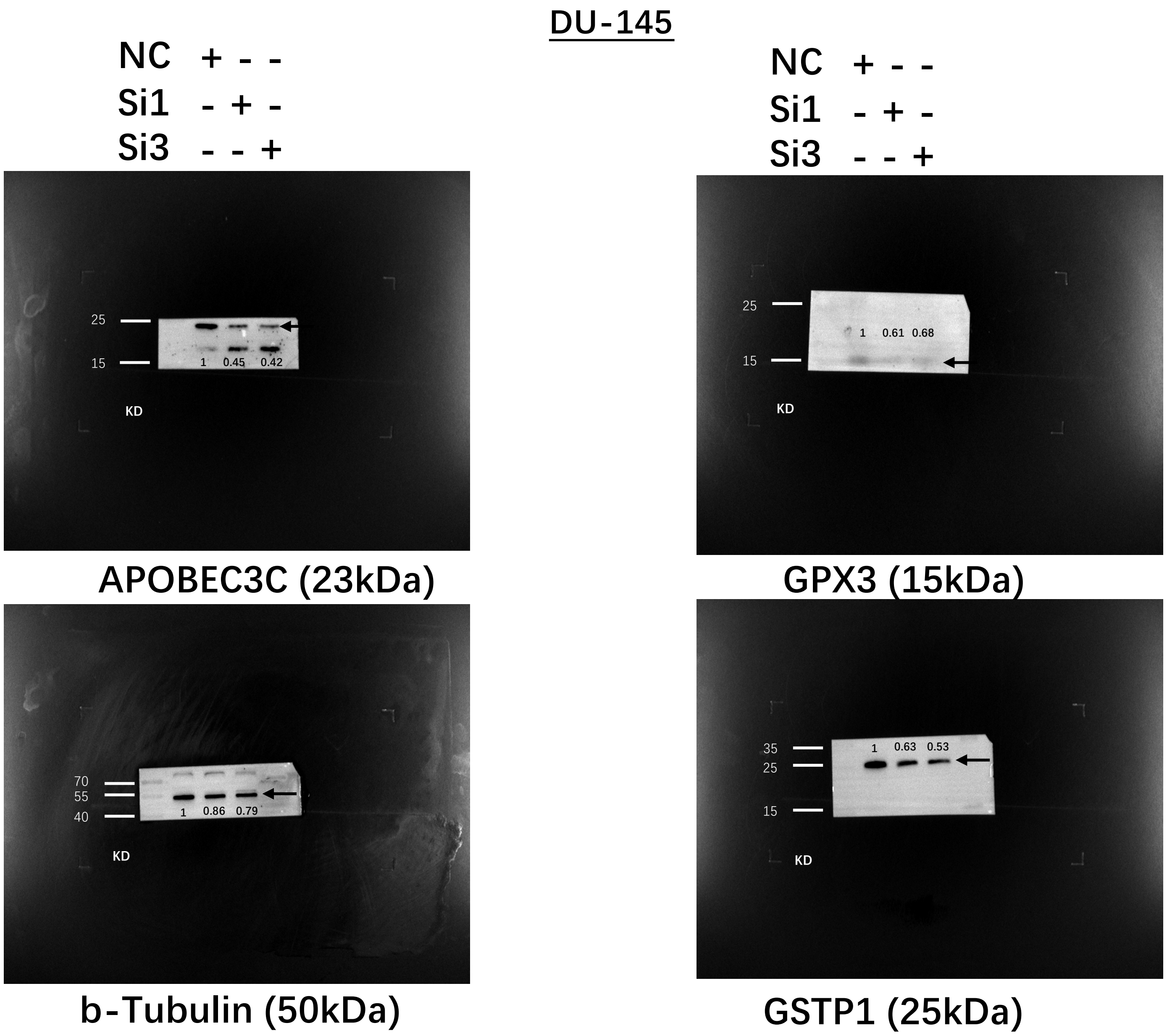
DU-145
NC + - -
Si1 - + -
Si3 - - +
25
15
1 0.45 0.42
KD
APOBEC3C (23kDa)
NC + - -
Si1 - + -
Si3 - - +
25
15
1 0.61 0.68
KD
GPX3 (15kDa)
70
55
40
1 0.86 0.79
KD
b-Tubulin (50kDa)
35
25
15
1 0.63 0.53
KD
GSTP1 (25kDa)

Fig.9. A

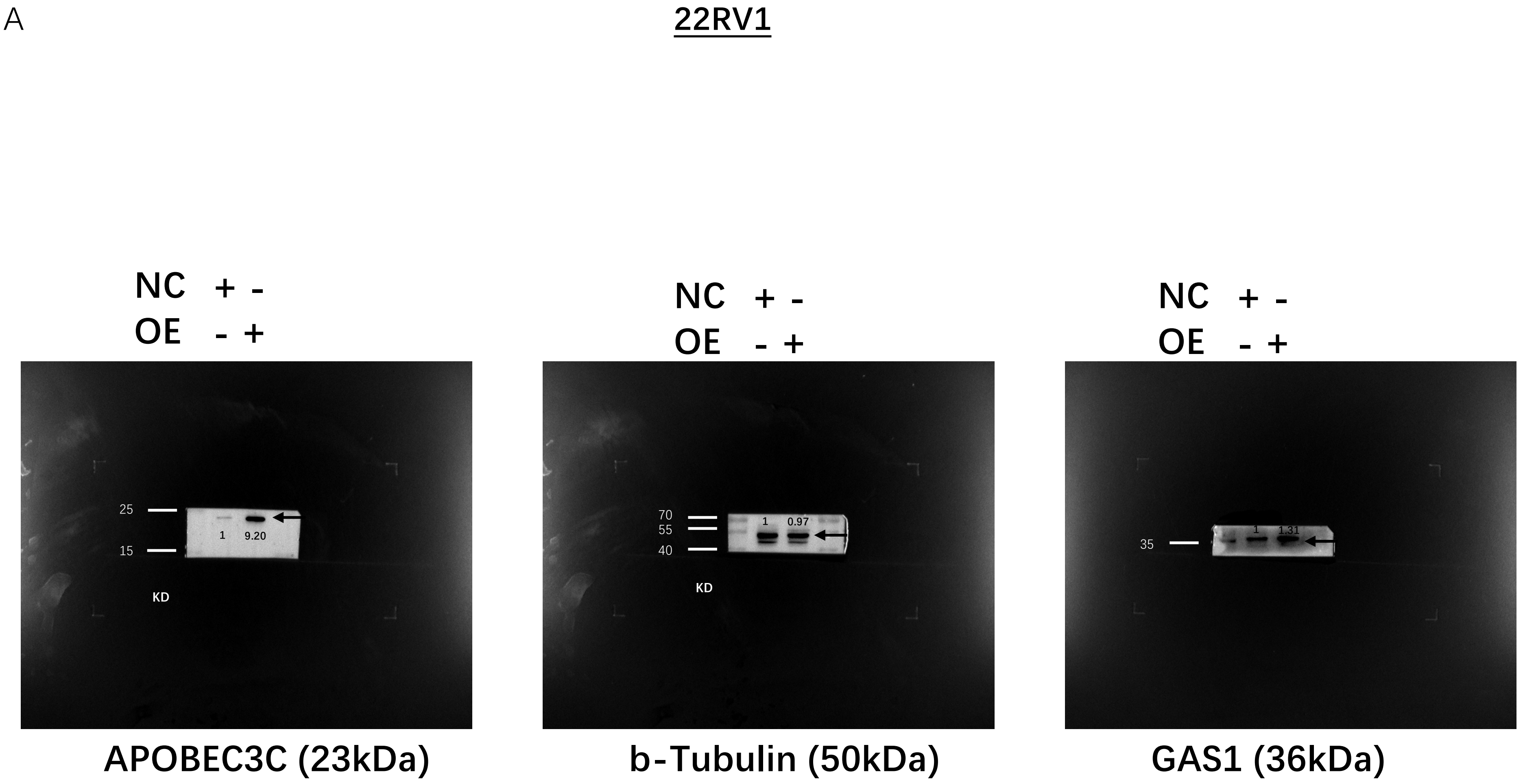
22RV1
NC + -
OE - +
25
15
KD
1
9.20
APOBEC3C (23kDa)
NC + -
OE - +
70
55
40
KD
1
0.97
b-Tubulin (50kDa)
NC + -
OE - +
35
1
1.31
GAS1 (36kDa)

Fig.9. A

DU-145

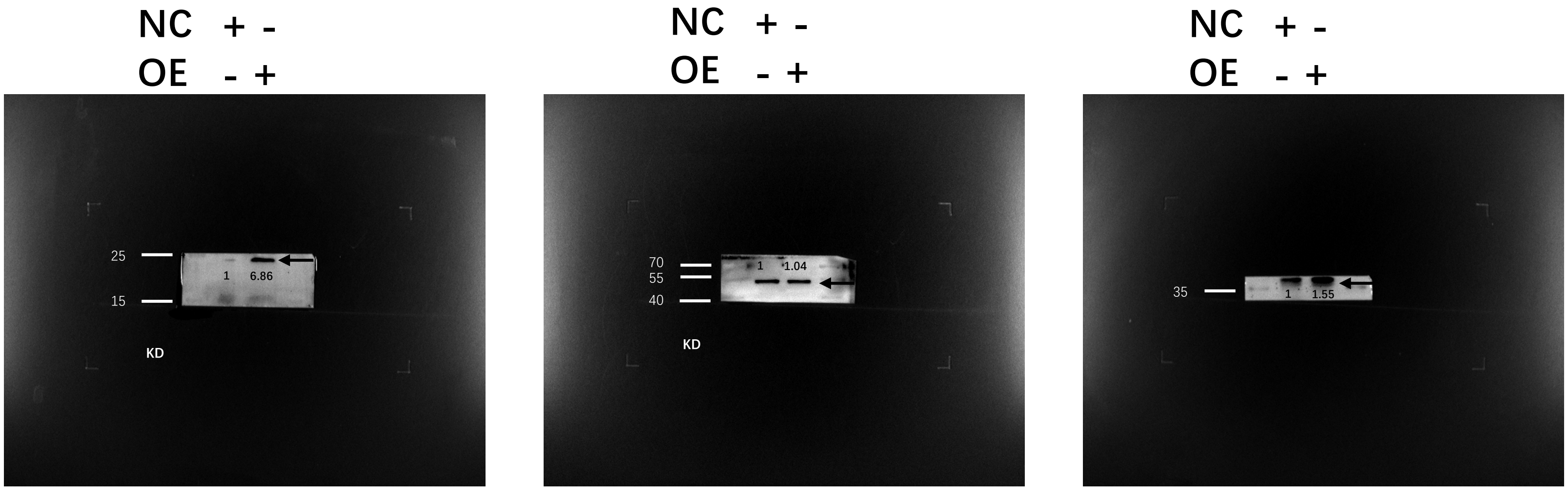

APOBEC3C (23kDa) b-Tubulin (50kDa) GAS1 (36kDa)

Fig.9. C

PC-3

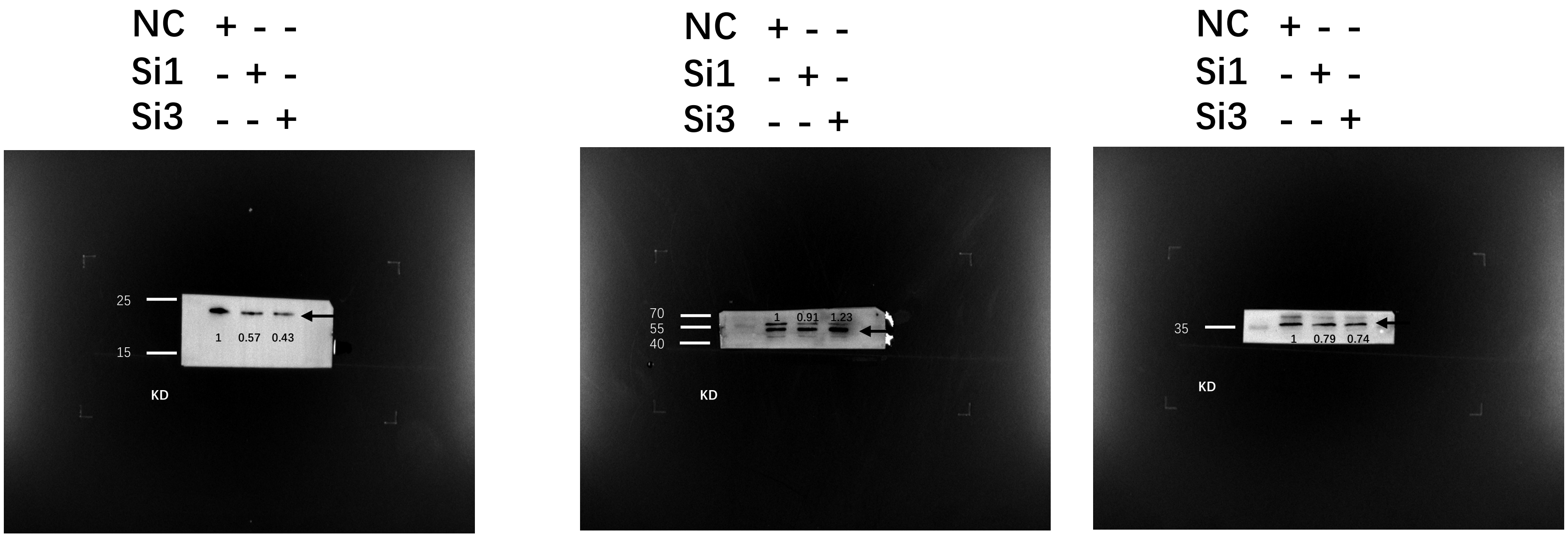

APOBEC3C (23kDa)   b-Tubulin (50kDa)   GAS1 (36kDa)

Fig.9. E

DU-145

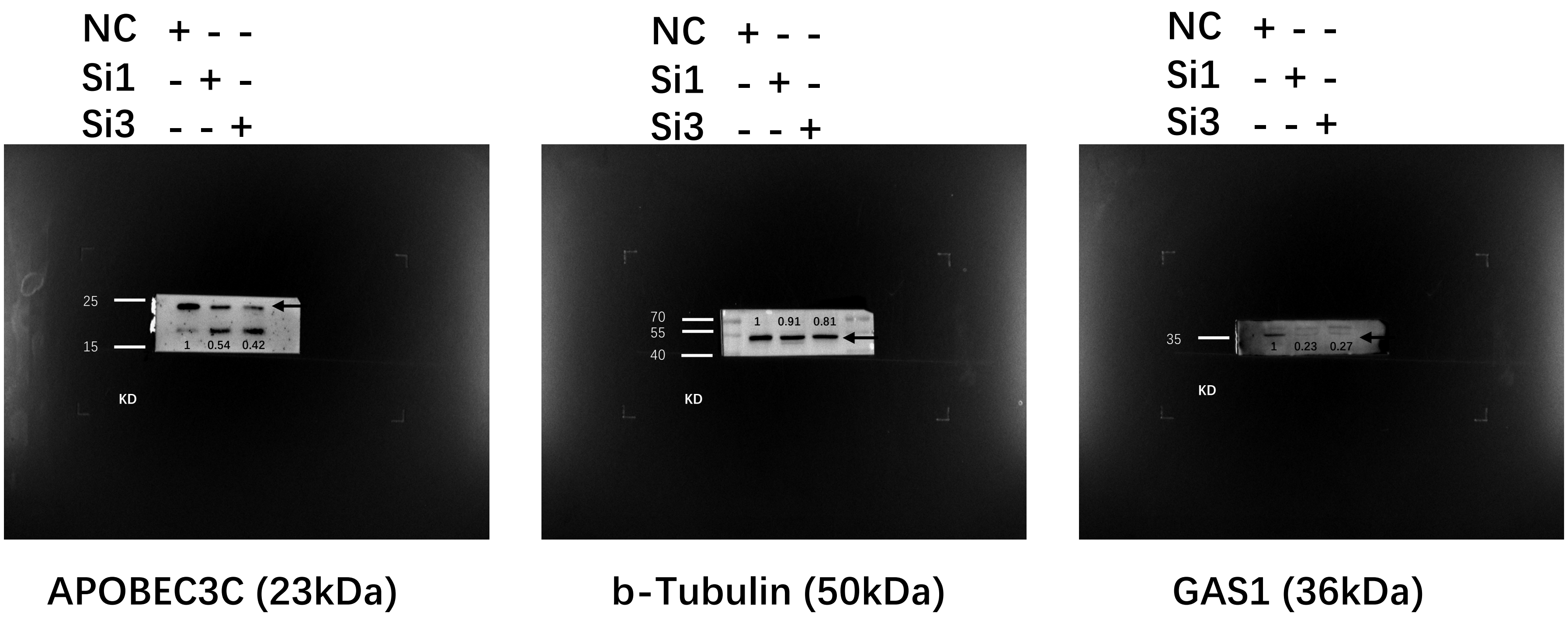
NC + - -
Si1 - + -
Si3 - - +
25
15
1 0.54 0.42
KD
APOBEC3C (23kDa)
NC + - -
Si1 - + -
Si3 - - +
70
55
40
1 0.91 0.81
KD
b-Tubulin (50kDa)
NC + - -
Si1 - + -
Si3 - - +
35
1 0.23 0.27
KD
GAS1 (36kDa)

22RV1

Supplementary
Fig.3. C

NC + -
OE - +

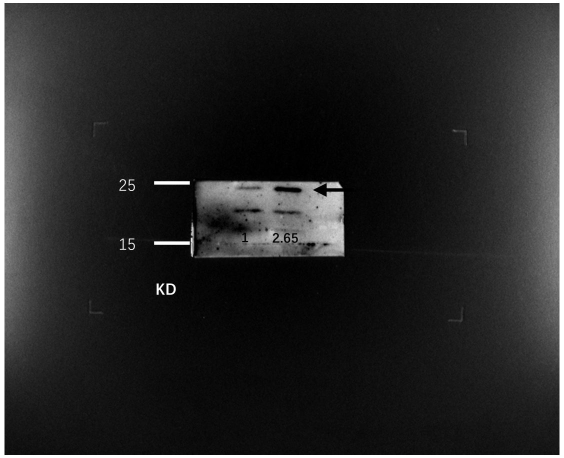

APOBEC3C (23kDa)

NC + -
OE - +

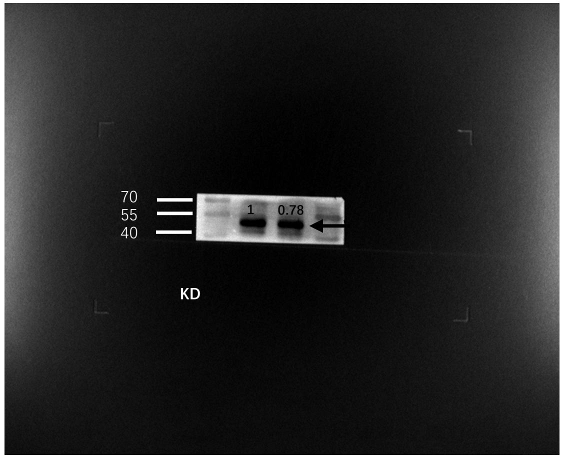

b-Tubulin (50kDa)

NC + -
OE - +

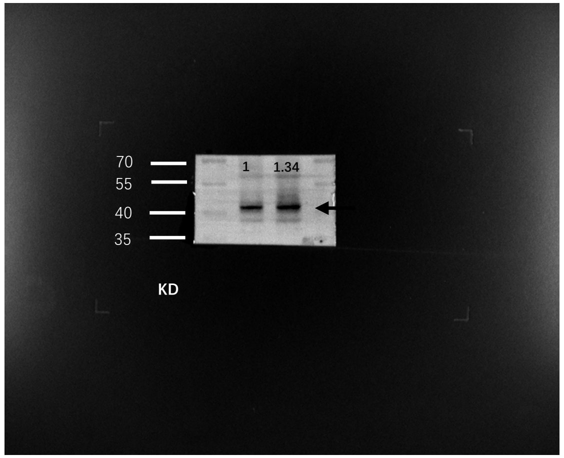

CD40 (40kDa)

DU-145

Supplementary
Fig.3. C

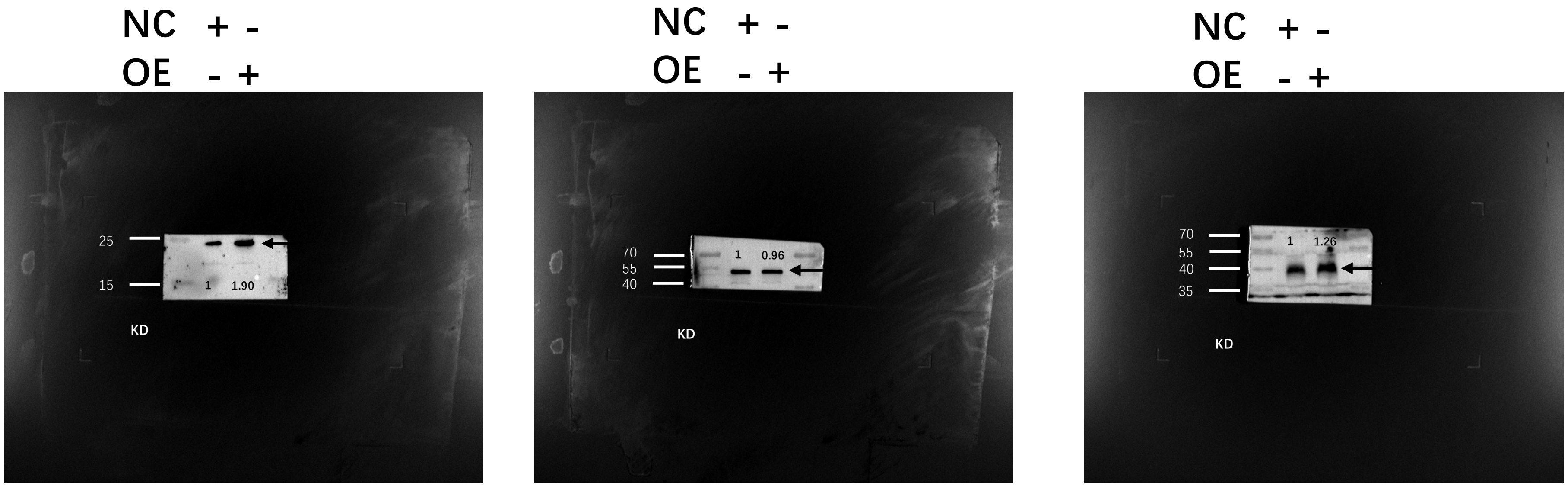

APOBEC3C (23kDa) b-Tubulin (50kDa) CD40 (40kDa)

DU-145

Supplementary
Fig.3. E

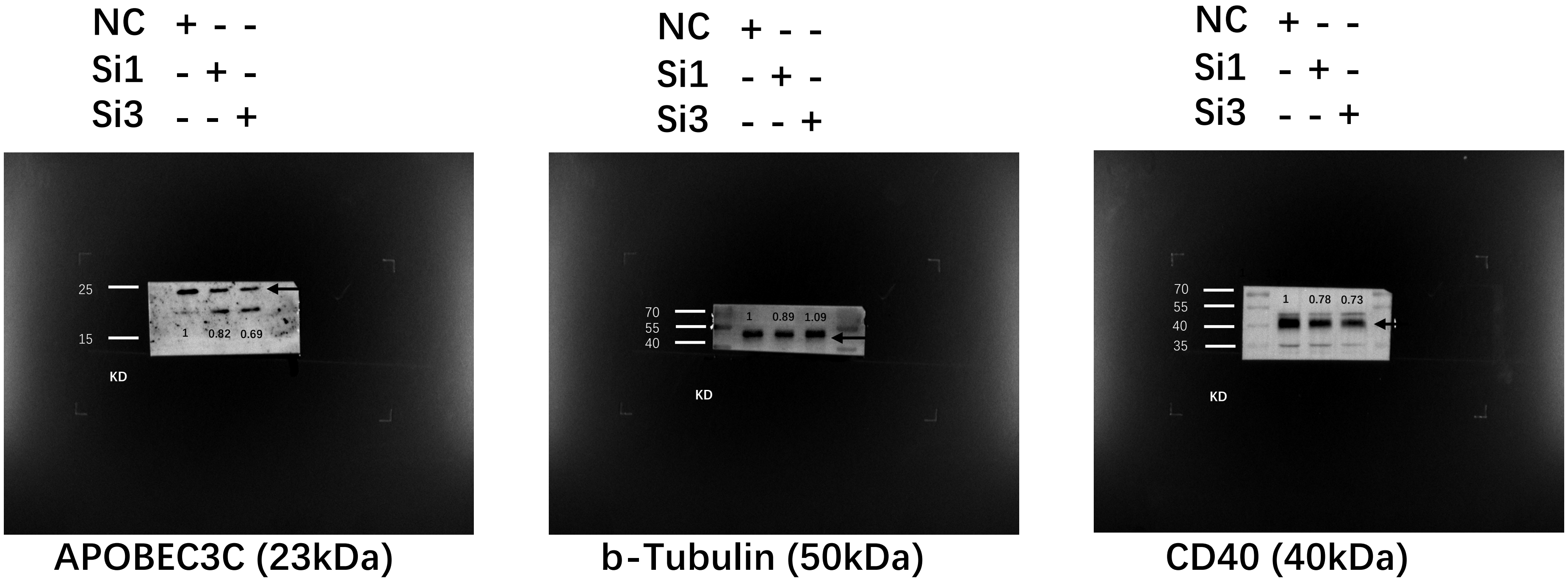

PC-3

Supplementary
Fig.3. G

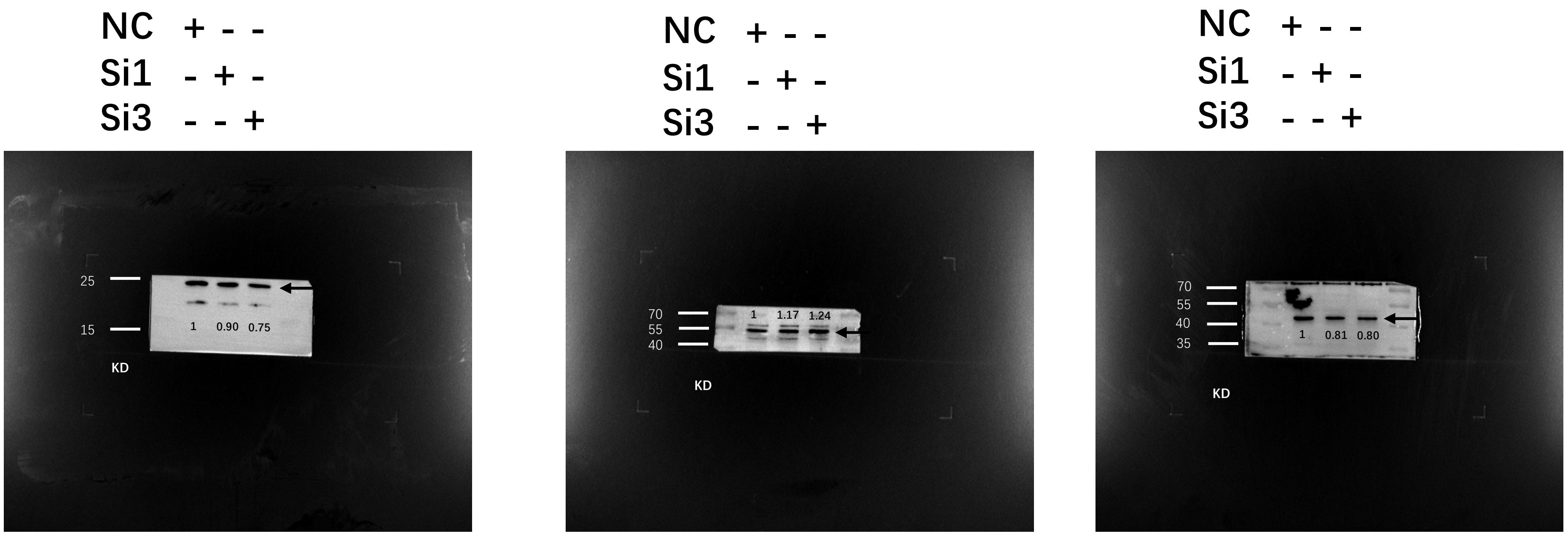

APOBEC3C (23kDa) b-Tubulin (50kDa) CD40 (40kDa)
