# Supplementary figures and images for: APOBEC3C Suppresses Prostate Cancer by Regulating Key Molecules Involved in Cellular Inflammation, Cell Cycle Arrest, and DNA Damage Response
Source: Cancers (Basel). 2026 Jan 3;18(1):170. doi: 10.3390/cancers18010170 (PMC12785094; doi:10.3390/cancers18010170)

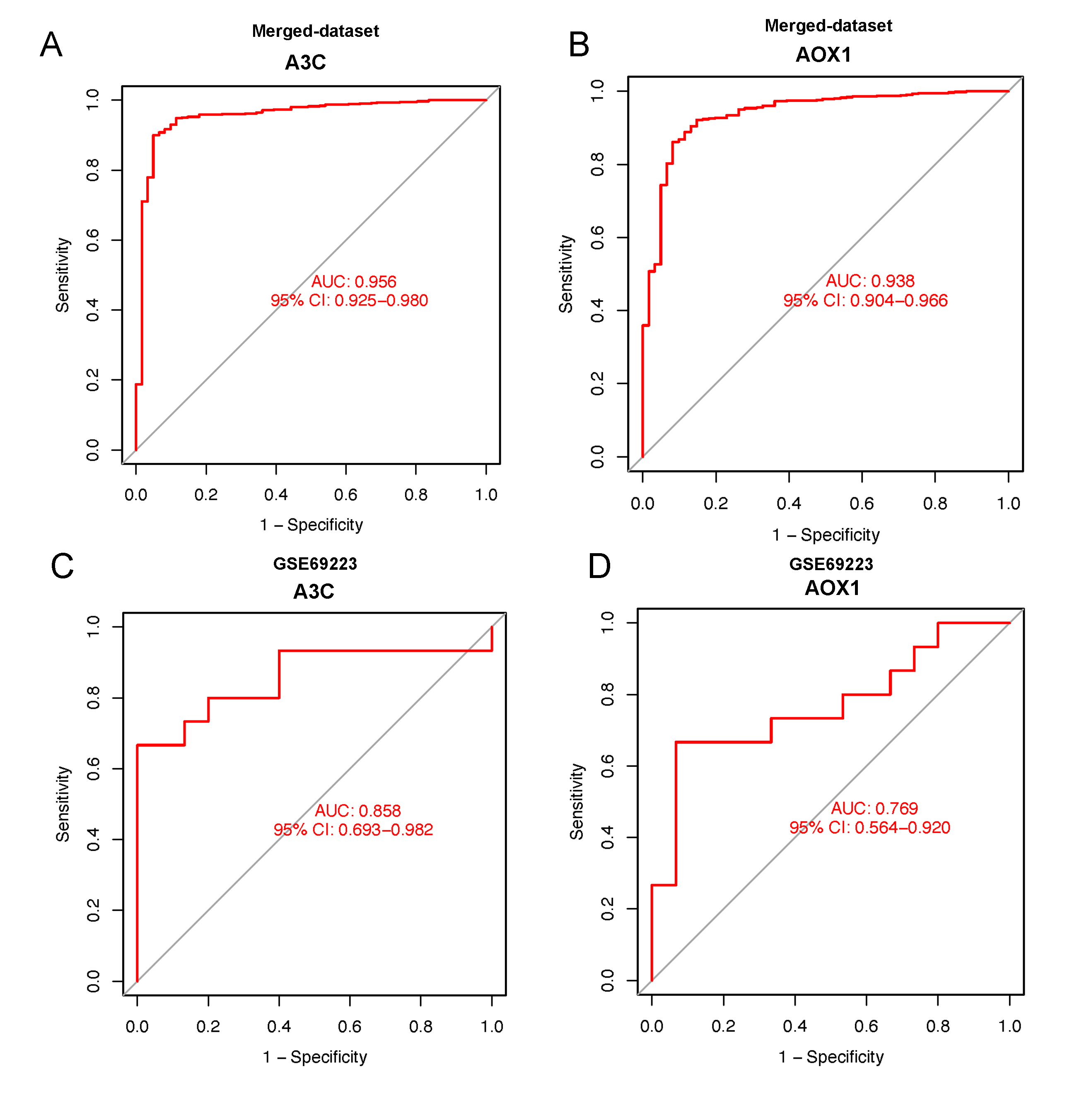

Supplement: Supplementary file 1 [file cancers-18-00170-s001.zip › Supplementary Figure S1.tif]

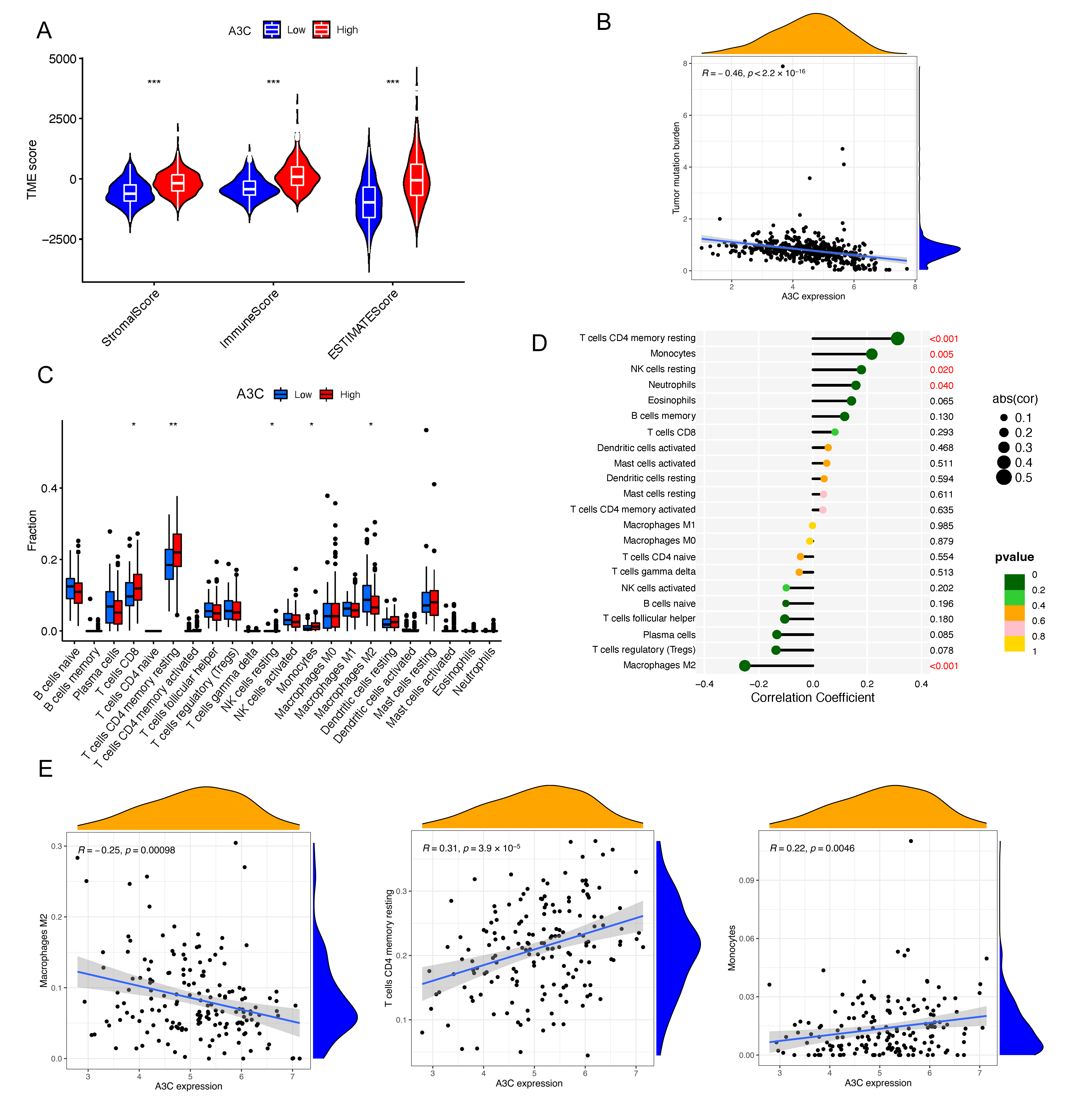

Supplement: Supplementary file 1 [file cancers-18-00170-s001.zip › Supplementary Figure S2.tif]

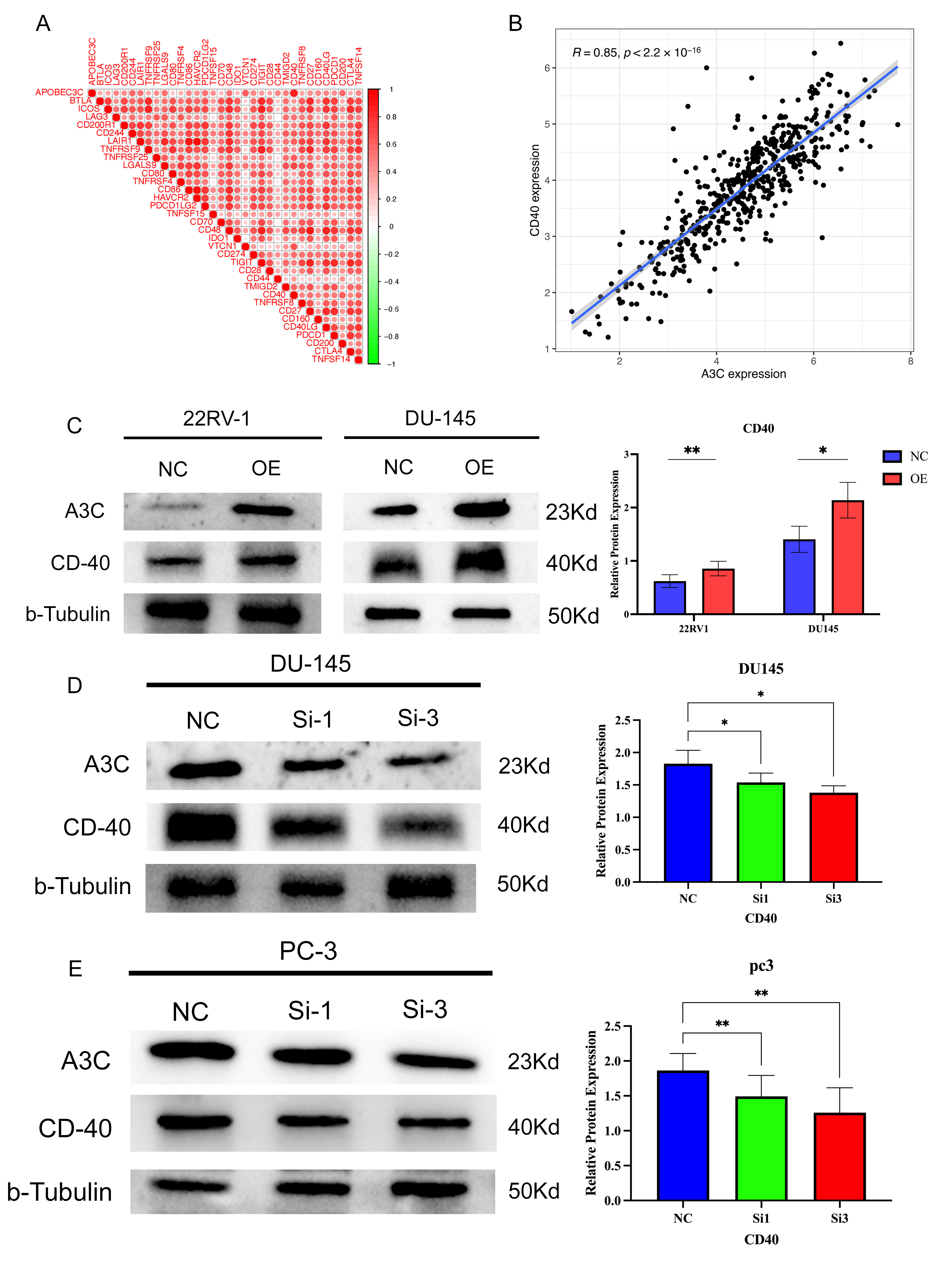

Supplement: Supplementary file 1 [file cancers-18-00170-s001.zip › Supplementary Figure S3.tif]

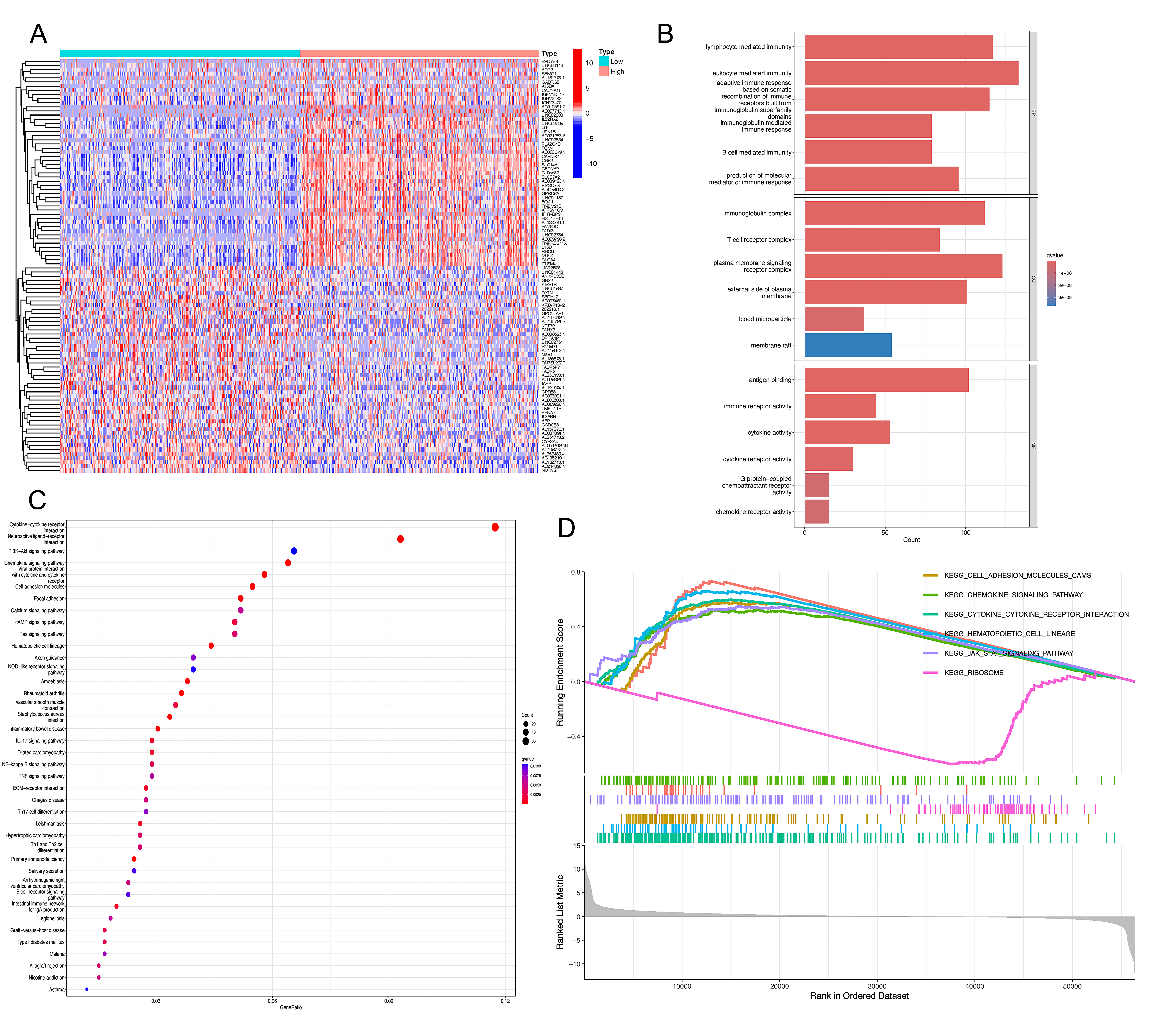

Supplement: Supplementary file 1 [file cancers-18-00170-s001.zip › Supplementary Figure S4.tif]

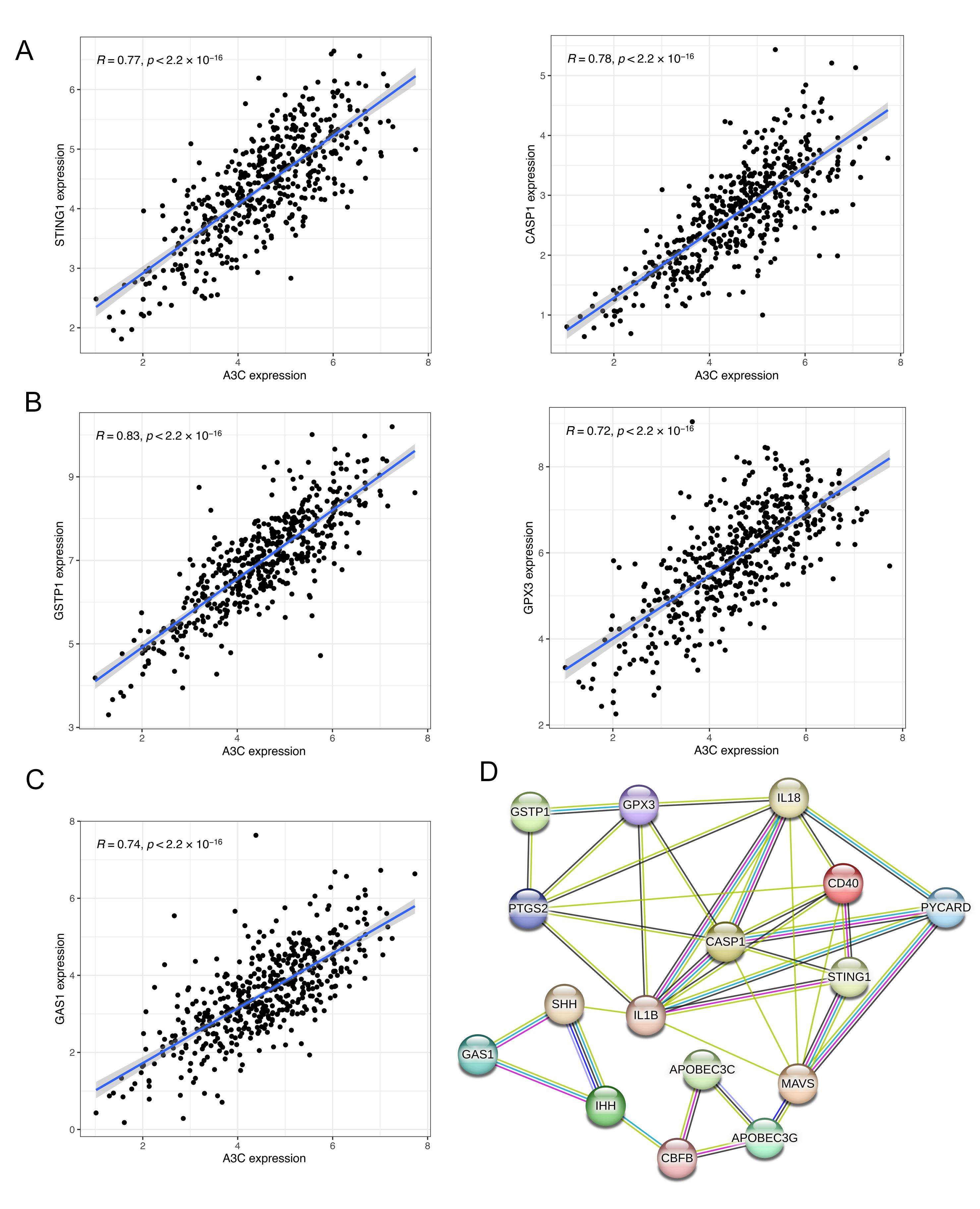

Supplement: Supplementary file 1 [file cancers-18-00170-s001.zip › Supplementary Figure S5.tif]
